# Variable-heavy (VH) families influencing IgA1&2 engagement to the antigen, FcαRI and superantigen proteins G, A, and L

**DOI:** 10.1038/s41598-022-10388-5

**Published:** 2022-04-20

**Authors:** Wei-Li Ling, Chinh Tran-To Su, Wai-Heng Lua, Joshua Yi Yeo, Jun-Jie Poh, Yuen-Ling Ng, Anil Wipat, Samuel Ken-En Gan

**Affiliations:** 1 Antibody & Product Development Lab, Experimental Drug Development Centre, Bioinformatics Institute, Agency for Science, Technology and Research (A*STAR), Singapore, Singapore; 2 Newcastle University Singapore, Singapore, Singapore; 3 School of Computing, Newcastle University, Newcastle upon Tyne, UK; 4 James Cook University, Singapore, Singapore; 5 Zhejiang Bioinformatics International Science and Technology Cooperation Center, Wenzhou-Kean University, Wenzhou, Zhejiang Province China; 6 Wenzhou Municipal Key Lab of Applied Biomedical and Biopharmaceutical Informatics, Wenzhou-Kean University, Wenzhou, Zhejiang Province China

**Keywords:** Cancer, Immunology, Molecular biology

## Abstract

Interest in IgA as an alternative antibody format has increased over the years with much remaining to be investigated in relation to interactions with immune cells. Considering the recent whole antibody investigations showing significant distal effects between the variable (V) and constant (C)- regions that can be mitigated by the hinge regions of both human IgA subtypes A1 and A2, we performed an in-depth mechanistic investigation using a panel of 28 IgA1s and A2s of both Trastuzumab and Pertuzumab models. FcαRI binding were found to be mitigated by the differing glycosylation patterns in IgA1 and 2 with contributions from the CDRs. On their interactions with antigen-Her2 and superantigens PpL, SpG and SpA, PpL was found to sterically hinder Her2 antigen binding with unexpected findings of IgAs binding SpG at the CH2-3 region alongside SpA interacting with IgAs at the CH1. Although the VH3 framework (FWR) is commonly used in CDR grafting, we found the VH1 framework (FWR) to be a possible alternative when grafting IgA1 and 2 owing to its stronger binding to antigen Her2 and weaker interactions to superantigen Protein L and A. These findings lay the foundation to understanding the interactions between IgAs and microbial superantigens, and also guide the engineering of IgAs for future antibody applications and targeting of superantigen-producing microbes.

## Introduction

As the highest produced antibody (66 mg/ mL per day) making up 75% of antibodies in mucosal areas^1,2^, IgA plays a key role in protecting a vast surface area of ~ 400 m^2^^1^ of the body, including the respiratory and gastrointestinal tracts^3^. Beyond its role in mucosal immunity, IgA confers passive immunity through maternal transfer to newborns via breastfeeding such as in the case of SARS-COV-2 antibodies^4^. There has been increasing interest in IgA^5^ as novel therapeutics given its efficiency to elicit antibody dependent cell-mediated cytotoxicity (ADCC)^6–8^, antibody dependent cell-mediated phagocytosis (ADCP)^9^, secretion of myeloperoxidase^10^, reactive oxygen species (ROS) production^10^ and neutrophil extracellular traps release (NETosis)^11,12^ against both infectious diseases^13–17^ and ductal tumors^5,18^ in oncology.

The microbial flora in mucosal areas^19^ where IgA is commonly found, e.g. *Staphylococcus aureus*, secrete superantigens that bind antibodies^20^ which prevents IgA-FcαRI interactions and serum killing of bacteria^21^, increasing susceptibility to septicemia (one of leading causes of death)^22^. These superantigens were also speculated to be linked to glomerulonephritis^23,24^, mimicking IgA nephropathy^25,26^, having clinical pathogenesis, thus requiring the engineering of future therapeutic IgAs resistance against such effects. Interventions and mitigations to such clinical pathogenesis and biologics engineering require the in-depth mechanistic understanding to accommodate the desired superantigen-based purification commonly used in biologics manufacturing^27^. While Proteins G (SpG), A (SpA), and L (PpL) are commonly used for purifying IgG therapeutics, the matrix choices for IgA purification are typically that of Peptide M^28^, IgA-binding protein, Jacalin^27^, and recently, the new Protein A/G^29^.

The two subclasses of IgAs: IgA1 and IgA2, differ in post-translational glycosylation patterns^30–33^ and at the hinge connecting the Cα1 and Cα2 of the heavy chains. Compared to IgA2, IgA1 has a longer hinge, allowing it to be more flexible for better accessibility to bind antigens^34^. IgAs exist in monomeric; dimeric; and secretory forms, with the monomeric forms predominantly found in serum as IgA1^5^ to bind FcαRI (CD89)^35–37^ on myeloid lineage cells^38–41^ for immune activation^42^. The dimeric forms, existing predominantly as two monomeric IgA2s conjoined at the tails of their constant (C) regions by the 16 kDa J-chain protein^43,44^, are primarily found at the mucosal areas. This binding of the dimeric IgA2 to the polymeric Ig receptor (plgR) expressed on the basolateral side of epithelial cells allows its translocation to the lumen after attachment and cleavage of the extracellular part of plgR known as secretory component (SC). These translocated pIgR-dimeric IgA2s are called secretory IgA (sIgA) where the SC stabilizes the complex, but also sterically hinders FcαRI binding due to the overlapping binding sites^44–46^.

IgAs differ from IgGs at the hinge region, glycosylation pattern and constant chain sequences which affect antibody accessibility to epitopes (discussed in this review^47^) and antigen engagement^48^. Given that mutual distal effects were reported between the variable (V)- to constant (C)-regions in IgGs^49^ and IgE^50^ where changes in V-regions could modulate interactions with their respective isotype FcRs, and reverse effects simultaneously reported for IgAs where C-region mutations mitigated antigen binding^48,51^, we sought to investigate the effect on IgA. Bearing in mind the contrasting hinges between the two IgA isotypes, the bi-directional effects would be expectedly distinct. Thus, for a more systematically holistic investigation, we grafted Pertuzumab and Trastuzumab CDRs onto seven human heavy chain V-region heavy chain families (VH1-VH7) of IgA1 and IgA2 C-regions^48^ to study effects on both antigen (Her2) and receptor (FcαRI, given reports of the heavy chain families being the major contributor of antigen specificity^49^). Since the IgA main receptor is FcαRI with its splice variants^52,53^, the IgA- FcαRI investigations would focus on monomeric IgAs. The in-depth understanding of this IgA-FcR mechanism by the various regions of IgA has clear implications in guiding biologics manufacturing, purification, and engineering for safety^54^, and also in contributing to the possible underlying mechanism of mucosal immunity, clinical glomerulonephritis and future mucosal vaccines against mucosal infectious diseases^55,56^.

## Results

### Bio-layer interferometry (BLI) measurements of PpL and anti-IgA immobilized Pertuzumab and Trastuzumab VH1-7 IgA1 and 2 variants to His-tagged Her2

To systematically study the effect of the VH framework (FWR) families on antigen binding by the IgAs, the various VH family variants were paired with their respective Pertuzumab/Trastuzumabκ Vκ1 light chain family and loaded on PpL and SA coupled anti-IgA sensors (See Supplementary Figs. S1–7 & S12 for loading graphs).

The Pertuzumab IgA1 and IgA2 variants showed a range of dissociation equilibrium constants (KD) from 7.25 to 50.55 × 10^–9^ M (Table 1). Among the 14 variants, PVH4-IgA2 was found to be the best binder (by KD) while PVH5-IgA1 was the weakest binder. Between the two antibody subtypes, there was a trend within the same VH family that IgA2 had generally lower(better) KD compared to IgA1. VH1, 3 and 4 showed KD values toward the Her2 antigen at the range of ~ 7 to 9 × 10^–9^ M while VH2 and 7 had moderate KDs with range of ~ 10 to 17 × 10^–9^ M with VH5 and 6 having the weakest (highest) KDs of ~ 28 and 50 × 10^–9^ M. The KDs differences of better binders were due to the lower rates of dissociation (kd) while moderate and weaker binders had higher kd rates (Supplementary Table S1).

**Table 1:** BLI measurements of PpL and anti-IgA biosensor immobilized Pertuzumab (PVH) and Trastuzumab (HVH) 1–7 IgA1 and IgA2 interaction to Her2. The KD values with standard error of each variant are shown accordingly. The KD differences between the two immobilization methods are shown in the last column with “x” indicating the fold differences. Poor response (PR) indicates that the antibody construct did not yield reliable ka and kd measurements (in triplicates) and NIL indicates the lack of data (PR) for comparison under Protein L immobilization. All measurements were performed in triplicates and rounded off to the nearest 2 decimal places. The ka and kd values are shown in Supplementary Table S1.

Binding measurement to Her2			
Construct	Protein L Immobilization	Anti-IgA Immobilization	Immobilization KD Differences
	KD (10^–9^)	KD (10^–9^)	KD
PVH1-IgA1	7.53 ± 0.03	0.23 ± 0.01	~ 32.7x
PVH2-IgA1	13.43 ± 0.47	2.90 ± 0.06	~ 4.6x
PVH3-IgA1	9.86 ± 0.05	0.68 ± 0.01	~ 14.5x
PVH4-IgA1	8.95 ± 0.04	0.58 ± 0.01	~ 15.4x
PVH5-IgA1	50.55 ± 0.81	7.09 ± 0.11	~ 7.1x
PVH6-IgA1	36.62 ± 0.16	6.57 ± 0.04	~ 5.5x
PVH7-IgA1	17.60 ± 0.07	2.67 ± 0.02	~ 6.5x
PVH1-IgA2	7.64 ± 0.05	0.47 ± 0.01	~ 16.2x
PVH2-IgA2	10.37 ± 0.29	3.45 ± 0.06	~ 3.0x
PVH3-IgA2	8.40 ± 0.05	0.85 ± 0.01	~ 9.8x
PVH4-IgA2	7.25 ± 0.04	0.95 ± 0.01	~ 7.6x
PVH5-IgA2	32.29 ± 0.53	7.21 ± 0.10	~ 4.4x
PVH6-IgA2	28.76 ± 0.11	6.82 ± 0.04	~ 4.2x
PVH7-IgA2	16.24 ± 0.08	2.95 ± 0.02	~ 5.5x
HVH1-IgA1	1.16 ± 0.02	0.10 ± 0.01	~ 11.6x
HVH2-IgA1	PR	0.83 ± 0.01	NIL
HVH3-IgA1	5.60 ± 0.04	0.35 ± 0.01	~ 16.0x
HVH4-IgA1	PR	0.50 ± 0.01	NIL
HVH5-IgA1	4.85 ± 0.04	0.62 ± 0.01	~ 7.8x
HVH6-IgA1	4.52 ± 0.04	0.43 ± 0.01	~ 10.5x
HVH7-IgA1	5.72 ± 0.04	0.57 ± 0.01	~ 10.0x
HVH1-IgA2	PR	0.61 ± 0.01	NIL
HVH2-IgA2	PR	1.02 ± 0.02	NIL
HVH3-IgA2	5.85 ± 0.05	0.34 ± 0.01	~ 17.2x
HVH4-IgA2	PR	0.99 ± 0.02	NIL
HVH5-IgA2	5.23 ± 0.05	0.57 ± 0.01	~ 9.1x
HVH6-IgA2	PR	0.29 ± 0.01	NIL
HVH7-IgA2	5.53 ± 0.05	0.59 ± 0.01	~ 9.3x

To cross validate Pertuzumab CDR grafting effects on IgA1 and 2, the highly similar Trastuzumab with almost identical V-region FWRs was chosen for comparison.

Trastuzumab IgAs variants were observed to differ from Pertuzumab IgAs variants with a narrower range of KD values (Table 1) between 1.16 and 5.85 × 10^–9^ M. The best binder among the 14 Trastuzumab variant is HVH1-IgA1 while the weakest binder with a measurable reading is HVH3-IgA2. Several of the Trastuzumab IgA variants were below the detection limits of BLI: HVH2, HVH4-IgA1, HVH1, HVH2, HVH4 and HVH6-IgA2. The trend between the A1 and A2 subclasses were reversed compared to Pertuzumab with Trastuzumab IgA1s having better KDs than its counterpart IgA2s of the same VH family. Unlike the Pertuzumab IgA variants with a spectrum in Her2 binding, Trastuzumab IgAs were more polarized with variants found at the extremes of binding range.

To rule out possible interference, avidity and protein orientation capture effects caused by PpL capture of the antibodies, we also implemented biotinylated anti-IgAs bound onto SA biosensors to immobilize the IgA variants at the Fc region for cross-validation antigen binding measurements.

The KD range of Pertuzumab IgAs binding Her2 (Table 1) was between 0.23 and 7.21 × 10^–9^ M which translates to ~ 3.0–32.7 times (Table 1) that of measurements using PpL, albeit with similar trends. VH1, 3 and 4 were the better binders with lower KDs of ~ 0.2–0.9 × 10^–9^ M, VH2 and 7 with moderate KDs at ~ 2.6–3.4 × 10^–9^ M, and VH5 and 6 as the weakest binders with KD values at ~ 6.5–7.2 × 10^–9^ M. Within anti-IgA immobilization, Pertuzumab IgA1s showed a lower (better) KDs than IgA2 of the same VH family FWRs which showed a reverse trend of IgA1 and 2 from PpL-based measurements.

Using anti-IgA immobilize at the Fc region for the Trastuzumab IgAs, the KD values increased by ~ 7.8–17.2 times compared to that of using PpL as was observed for the Pertuzumab IgAs (Table 1).

### BLI measurements of NTA-Ni immobilized FcαRI interacting with Pertuzumab and Trastuzumab VH1-7 of IgA1 and 2

To investigate the effects of the VH families on FcαRI engagement by the IgAs, recombinant His-tagged FcαRI was bound to NTA-Ni biosensors prior to measurements (See Supplementary Figs. S8–S11 for loading graphs).

The interaction (KD) with Pertuzumab IgAs ranged from 0.84 to 3.26 × 10^–8^ M (Table 2) with the best binder as VH5-IgA1 and the weakest binder as VH2-IgA2. Both VH5 IgA1 and 2 are strong binders to FcαRI at KDs 0.84 and 0.97 × 10^–8^ M with kd values of 3.94 and 4.41 × 10^–4^ 1/s respectively while the rest of the VH families (VH1, 2, 3, 4, 6 and 7) showed weak binding with high KDs of 1.85 to 3.26 × 10^–8^ M. Comparing the subtypes, IgA1s bound better to FcαRI than IgA2s of the same VH families.

**Table 2:** BLI measurements of NTA-Ni biosensor immobilized FcαRI interaction to Pertuzumab (PVH) and Trastuzumab (HVH) 1–7 IgA1 and IgA2. The KD, ka and kd values with standard error of each variant are shown accordingly. Poor response (PR) indicates that the antibody construct did not yield reliable ka and kd measurements (in triplicates). All measurements were performed in triplicates and rounded off to the nearest 2 decimal places.

Binding measurement to FcαRI			
Construct	NTA-Ni immobilization		
	KD (10^–8^)	ka (10^4^)	kd (10^–4^)
PVH1-IgA1	2.86 ± 0.05	10.70 ± 0.18	30.07 ± 0.16
PVH2-IgA1	3.02 ± 0.05	9.82 ± 0.15	27.86 ± 0.13
PVH3-IgA1	2.18 ± 0.03	11.10 ± 0.16	23.92 ± 0.12
PVH4-IgA1	2.33 ± 0.03	10.26 ± 0.15	23.72 ± 0.13
PVH5-IgA1	0.84 ± 0.01	4.76 ± 0.03	3.94 ± 0.03
PVH6-IgA1	2.18 ± 0.03	9.03 ± 0.11	19.53 ± 0.09
PVH7-IgA1	1.93 ± 0.03	12.16 ± 0.17	23.17 ± 0.12
PVH1-IgA2	2.38 ± 0.04	9.35 ± 0.12	21.47 ± 0.11
PVH2-IgA2	3.26 ± 0.05	5.88 ± 0.08	17.00 ± 0.09
PVH3-IgA2	1.85 ± 0.02	9.00 ± 0.10	16.03 ± 0.07
PVH4-IgA2	2.52 ± 0.03	7.60 ± 0.10	18.54 ± 0.09
PVH5-IgA2	0.97 ± 0.01	4.71 ± 0.04	4.41 ± 0.04
PVH6-IgA2	2.57 ± 0.03	5.84 ± 0.07	14.45 ± 0.08
PVH7-IgA2	1.94 ± 0.02	9.27 ± 0.10	17.74 ± 0.08
HVH1-IgA1	3.40 ± 0.05	2.55 ± 0.03	8.65 ± 0.05
HVH2-IgA1	PR		
HVH3-IgA1	1.01 ± 0.01	8.52 ± 0.10	8.47 ± 0.07
HVH4-IgA1	PR		
HVH5-IgA1	0.89 ± 0.01	7.03 ± 0.07	6.18 ± 0.05
HVH6-IgA1	2.16 ± 0.03	7.57 ± 0.10	16.20 ± 0.09
HVH7-IgA1	1.42 ± 0.02	8.13 ± 0.09	11.44 ± 0.07
HVH1-IgA2	3.15 ± 0.06	2.87 ± 0.05	9.59 ± 0.07
HVH2-IgA2	PR		
HVH3-IgA2	1.25 ± 0.02	8.00 ± 0.09	9.82 ± 0.07
HVH4-IgA2	PR		
HVH5-IgA2	0.79 ± 0.01	7.70 ± 0.07	6.08 ± 0.05
HVH6-IgA2	2.59 ± 0.04	3.26 ± 0.05	8.23 ± 0.06
HVH7-IgA2	1.16 ± 0.01	8.17 ± 0.08	9.30 ± 0.05

As shown in Table 2, the observed KD values for FcαRI interacting with Trastuzumab IgAs ranged from 0.79 to 3.40 × 10^–8^ M, with VH5-IgA2 as the best binder and VH1-IgA1 as the weakest binder. The interaction of FcαRI with Trastuzumab IgAs could be categorized into the strong binders (VH5 family) with KDs of 0.79 and 0.89 × 10^–8^ M with low kd values of 6.08–6.18 × 10^–4^ 1/s, with weak binders of VH1, 3, 6 and 7 families with KDs of 1.01–3.40 × 10^–8^ M and lastly, the non-binders: VH2 and 4. Unlike Pertuzumab variants, there was no noticeable trend of either Trastuzumab IgA1 or IgA2 within the same VH families.

### BLI measurements of immobilized proteins G, L and A interacting with Pertuzumab and Trastuzumab VH1-7 of IgA1 and 2

To investigate the effects of the VH families on IgAs interacting with antibody superantigens with relevance to mucosal immunity and antibody purification, interactions of the IgAs variants with superantigens: proteins G, L and A biosensors, were measured.

The KD range of binding to SpG (Table 3) was from 1.26 to 18.22 × 10^–9^ M with the best binder as HVH6-IgA2 and weakest binder as HVH3-IgA1. KD calculations of Trastuzumab variants were lower (better) than Pertuzumab variants and IgA1 generally had lower readings than the corresponding IgA2 of the same VH family. All the VH3 variants, regardless of IgA1 or 2 of Pertuzumab or Trastuzumab, had consistently higher KDs than the other VH variants within the groups tested due to the lower ka measurements (Supplementary Table S2).

**Table 3:** BLI measurements of immobilized proteins G, L and A interacting with Pertuzumab (PVH) and Trastuzumab (HVH) 1–7 IgA1 and IgA2. The KD values with standard error of each variant are shown accordingly. Poor response (PR) indicates that the antibody construct did not yield reliable ka and kd measurements (in triplicates). All measurements were performed in triplicates and rounded off to the nearest 2 decimal place. The ka and kd values are shown in Supplementary Table S2.

Binding measurement to superantigen			
Construct	Protein G immobilization	Protein L immobilization	Protein A immobilization
	KD (10^–9^)	KD (10^–10^)	KD (10^–8^)
PVH1-IgA1	5.25 ± 0.05	17.94 ± 0.39	PR
PVH2-IgA1	3.24 ± 0.03	20.82 ± 0.32	22.74 ± 0.46
PVH3-IgA1	9.49 ± 0.13	7.95 ± 0.37	6.17 ± 0.13
PVH4-IgA1	3.45 ± 0.03	14.45 ± 0.32	21.86 ± 0.45
PVH5-IgA1	2.78 ± 0.03	28.92 ± 0.31	22.37 ± 0.47
PVH6-IgA1	2.60 ± 0.03	24.70 ± 0.29	21.03 ± 0.41
PVH7-IgA1	4.25 ± 0.04	22.64 ± 0.33	PR
PVH1-IgA2	7.62 ± 0.06	11.56 ± 0.25	PR
PVH2-IgA2	3.15 ± 0.02	31.00 ± 0.18	27.42 ± 0.78
PVH3-IgA2	14.45 ± 0.16	5.21 ± 0.24	6.10 ± 0.10
PVH4-IgA2	6.52 ± 0.05	11.40 ± 0.25	PR
PVH5-IgA2	3.77 ± 0.02	22.66 ± 0.18	75.49 ± 6.00
PVH6-IgA2	3.70 ± 0.02	21.69 ± 0.18	79.10 ± 10.68
PVH7-IgA2	9.87 ± 0.10	12.93 ± 0.27	PR
HVH1-IgA1	1.98 ± 0.04	48.38 ± 0.23	11.20 ± 0.11
HVH2-IgA1	1.59 ± 0.04	88.53 ± 0.41	3.70 ± 0.02
HVH3-IgA1	18.22 ± 0.28	1.35 ± 0.39	0.47 ± 0.01
HVH4-IgA1	1.52 ± 0.04	51.31 ± 0.42	PR
HVH5-IgA1	3.19 ± 0.04	10.82 ± 0.47	16.50 ± 0.33
HVH6-IgA1	1.85 ± 0.04	18.60 ± 0.37	6.20 ± 0.10
HVH7-IgA1	2.52 ± 0.04	11.55 ± 0.42	3.43 ± 0.06
HVH1-IgA2	2.51 ± 0.06	47.05 ± 0.59	0.28 ± 0.01
HVH2-IgA2	1.70 ± 0.03	56.36 ± 0.45	0.84 ± 0.01
HVH3-IgA2	14.12 ± 0.24	0.07 ± 0.25	0.42 ± 0.01
HVH4-IgA2	1.79 ± 0.04	72.59 ± 0.42	7.77 ± 0.09
HVH5-IgA2	4.14 ± 0.04	1.91 ± 0.20	27.42 ± 0.91
HVH6-IgA2	1.26 ± 0.03	42.05 ± 0.25	5.02 ± 0.04
HVH7-IgA2	3.06 ± 0.03	7.06 ± 0.25	4.30 ± 0.08

On binding to PpL (Table 3), the KD values ranged from 0.07 to 88.53 × 10^–10^ M, with the best binder as HVH3-IgA2 and weakest binder as HVH2-IgA1. KD values of the Pertuzumab variants were narrower/more consistent (5.21–31.00 × 10^–10^ M) when compared to that of the Trastuzumab variants (0.07–88.53 × 10^–10^ M). All VH3 variants showed the best KD among its respective groups (Pertuzumab or Trastuzumab of IgA1 or IgA2) as determined by the low kd (Supplementary Table S2). Apart from the VH3s, Trastuzumab VH5 and VH7 of IgA2 are also strong binders with KD values of 1.91 and 7.06 × 10^–10^ M, respectively.

On binding to SpA (Table 3), the KD values ranged from 0.28 to 79.10 × 10^–8^ M with the best binder as HVH1-IgA2 and weakest binder as PVH6-IgA2. Trastuzumab variants generally interacted with SpA (as is so with SpG) better than Pertuzumab variants. The VH3 variants showed better ka measurements within the groups, especially the Trastuzumab variants (~ 37 × 10^4^) when compared to Pertuzumab variants (~ 3 × 10^4^). Both VH1 and 2 variants of Trastuzumab IgA2 were strong binders with KD values of 0.28 and 0.84 × 10^–8^ M respectively. It should be noted that their lower KD values were due to lower kd measurements rather than higher ka as was observed with the VH3 variants (Supplementary Table S2). The VH5 variants of both Trastuzumab IgA1 and 2 had weaker binding within the respective IgA families, and both VH5 and 6 of Pertuzumab IgA2 had the highest KD (weakest binding) within the subtypes. There were a few variants with no detectable interaction with SpA such as PVH1- and 7-IgA1, PVH1-, 4-, 7-IgA2 and HVH4-IgA1with no consistent trends by VH families.

## Discussion

We set out to holistically study the effects of VH families on antigen (Her2) and receptor FcαRI binding on both IgA1 and IgA2 that were highly similar in sequences with respect to the C-region, but have notable differences in glycosylation and at the hinge regions. We found that the VH1 and VH3 FWRs together with the avoidance of VH2 and VH4 during CDR grafting to be suitable for both IgA subtypes even though VH4 was found suitable for Pertuzumab but not Trastuzumab. While VH3 is the canonical FWR of choice in antibody humanization due to its better production^49,57^, VH1 was shown to be potentially more suitable for IgAs given that VH3 has the propensities to bind SpA^50^ and metals like nickel^58^, which are both potential superantigens at mucosal areas.

BLI testing of Pertuzumab and Trastuzumab IgA1s and 2s immobilized via PpL biosensors showed agreement with our previous work^48,51^, but the KD values of the same IgA1s and 2s immobilized via biotinylated anti-IgA sensors showed increased measurements across all variants including those initially below reliable detection limits using PpL (Table 1). These results suggest that the binding of PpL on the light chain of the antibody sterically hindered Her2 binding at the CDRs.

Analyzing the differences from the KD values of both the PpL and anti-IgA immobilized methods, superantigen PpL, produced by *Finegoldia magna* (previously known as *Peptostreptococcus magnus*), may potentially destabilize IgA antigen binding given the ~ 3.0–32.7 times difference across all VH families, subtypes and CDRs (Table 1). Given that *Finegoldia magna* is a common commensal of the genitourinary and gastrointestinal tracts^59^ where IgA is the dominant antibody isotype and that the microbe is a common contaminant in blood^59^, PpL binding to the majority Vκ population of human antibodies highlights its potential pathogenicity. With significant superantigen-like activation of B-cells^20^ and the dampening of antigen binding (shown here with Her2), or to cross-link Vκ IgEs on sensitized basophils and mast cells at the mucosal areas to cause inflammation^60–62^, toxic shock syndrome^63^ is also in the list of pathological effects. While IgA therapeutics are still in the early stages of development, antibody engineering^54^ precautions can be made against such potential microbiome interaction that can impact beyond IgAs to the other isotypes. Avoidance of such interactions would be important in mitigating unwanted side effects leading to expensive drug failures, especially during clinical trials.

Apart from superantigen PpL, the binding to SpG and SpA revealed surprising IgA interactions mitigated by the V-regions that are contrary to canonical textbook reports and the company product information sheets (Supplementary Table S3) of these two superantigens. Our panel showed clear binding of IgAs to both proteins G and A that is in part moderated by VH-regions. Non-VH3 Trastuzumab variants bound strongly to SpA, a finding in partial agreement to a previous ELISA study^64^. Since both SpG and SpA are produced by the groups C & G of *Streptococcus *spp.^65,66^ and *S. aureus*^67^, respectively, and that both are part of the normal human flora at mucosal areas and skin surfaces^68^, this finding expands the potential interaction to isotypes such as IgE^50^ present on mast cells^62^ to trigger allergic reactions. Apart from unraveling the possible mechanisms underlying microbiome-antibody interactions at the mucosal area, there is a clear need to exploit or mitigate the IgA-superantigen properties beyond therapeutics to that of diagnostics, given the interactions with IgGs^69,70^.

Although, SpA only binds to specific populations of IgA^64^ (later found to be those of the VH3 family^71^) and the absence of SpG binding to IgAs are canonical knowledge, our panel provided deeper insights to these reports. Here, we demonstrated that non-VH3 IgAs could bind SpA and that these VH3 effects were mitigated by different CDRs and the CH1 subclass. There was clear synergistic contribution from the VH-FWRs, CDRs and CH1 to SpA-IgA binding. The contribution of CDRs to VH3 was also in agreement with our previous work for Trastuzumab IgE^58^. Although SpG does not canonically bind IgA, our results showed such interaction, narrowing them to occur at the IgA Fc, particularly the CH2/CH3 regions, since the KDs were relatively constant, demonstrating the lack of VH families, CDRs and IgA subclass effects.

In its role to activate effector immune cells, IgA therapeutics has to engage FcαRI effectively. The constant regions are commonly ignored in antibody design and development in reductionist screening display methods^72^, yet they perform the main role of FcR binding and are influenced by the V-regions as shown from our previous study of IgEs to the FcεRIα^50,58^. This thus signals for the need of a whole-antibody approach during the early stages of antibody therapeutics development to avoid unwanted surprises. Within the VH families, VH5 IgAs showed the lowest KD (best interactions) to FcαRI for both Pertuzumab and Trastuzumab in agreement to their IgE counterparts with FcεRIα. Since VH5 is incidentally the biased VH in some allergic patients^73^, its propensity to engage FcεRIα stronger^50^ and bind metal like nickel^58^, has potential disease pathogenic significance given its ability for longer interaction (lowest kd) with FcαRI. With both IgEs and IgAs as mucosal antibodies and the possible class switching to IgA2 from IgE^74,75^, there is much to investigate on VH5 FWRs in the allergy pathogenesis.

With regards to IgA immobilization, the increased KDs when using anti-IgA Fc immobilization compared to PpL at the Vκ1 of ~ 3–32.7-fold difference clearly demostrate interference by PpL at the V-regions across the variants. These differences were narrowed to the kd in Pertuzumab IgAs and ka in Trastuzumab IgAs (Table 1) where PpL binding at Vκ1-FWR1^76^ sterically blocked Her2 engagement.

Contradicting Her2 binding trends between the Trastuzumab and Pertuzumab IgAs were likely due to the slightly different combination of CDRs affecting the flexibility of the IgAs conferred by the hinge^51^, as well as the varying distribution of glycans (as shown in our computational models in Supplementary Figs. S14A and S15A), and the different locations of their target epitopes on Her2 (Supplementary Fig. S13) affecting general accessibility of the interacting V-region residues^47,77^. There are two different Her2 epitopes with respect to Trastuzumab and Pertuzumab, where the Pertuzumab-binding epitope was buried deeper as compared to that of Trastuzumab (Supplementary Fig. S13). Hence, the H3 loop of the Pertuzumab CDRs might adopt a more flexible and accessible conformation for binding the less exposed Her2 epitope as compared to those of the Trastuzumab CDRs, a possibility illustrated by the differences observed for Trastuzumab and Pertuzumab IgMs and IgG1s^47,77^.

There are differences in structural arrangements between the Trastuzumab IgA1 and IgA2 from their different hinges connecting Cα1 and Cα2 domains and glycan attachments (e.g. only IgA1 has *O*-linked glycans at the hinge region whereas IgA2 has two additional *N*-linked glycans sites at N166 and N324) that can cause different FcαRI binding orientations between the two Trastuzumab IgA1 and IgA2 isotypes. To study this, we modelled selected VH families of both Pertuzumab and Trastuzumab IgAs and performed docking to FcαRI (Supplementary Figs. S14A and S15A and Supplementary 2 for the computational work) and found the hydrophobic packing core at Pertuzumab central IgA1 and IgA2 Fc to be maintained with SASA < 30% and 40% respectively on the active residues (Supplementary Figs. S14B and S15B). The occupancy of the modeled glycans near the FcαRI-binding regions was used to screen the docked FcαRI-bound Trastuzumab IgAs VH2 complexes (Supplementary Fig. 14C and 15C) and the FcαRI-Fcα interfaces of the Trastuzumab IgA2 VH4 complexes (Supplementary Fig. 15C) showed the importance of glycans in FcαRI binding at the C-region, supporting a previous study by Steffen et al*.*^78^. However, extended sampling of the glycan dynamics combining the hinge-inducing structural constraints on the full length IgAs models upon or prior to the FcαRI binding are essential in the future work given that there was structural shifts of a few hydrophobic residues from the hydrophobic core (e.g. A442 or F443) occurring in both the Trastuzumab IgA1/IgA2 and Pertuzumab IgA2 variants. These structural arrangements could have resulted from the induced intradomain Cα2-Cα3 motion constraints (but not limited to the modeling artifact) that might dampen FcαRI interactions (for more detailed explanation, refer to Supplementary Figs. S14 and S15).

Apart from the indirect impact of the hinge onto the intradomain between Cα2 and Cα3 as well as the motions of the Fab region upon FcαRI^36^ (Supplementary Fig. S16), we observed possible steric hindrance caused by the flexible motions of the C-terminal tail piece of the IgA1-Fc region. With these C-terminal segments forming two symmetrical unstructured tails in our IgA1 models (whereas together with the J-chains constituting stable β-sheets in dimeric IgA1, i.e. PDB: 7JG1), FcαRI targeting the hydrophobic core of the IgA1 C-region was hindered sterically. This obstruction is absent in the truncated Fc and FcαRI complex (1OW0 lacking the C-terminal tail piece) or alleviated in the docking attempts of the modified Trastuzumab IgA1 VH3 variant (with the C-terminal tail removed), i.e. similar FcαRI binding orientation as observed in the 1OW0 being reproduced in one of the resulting docked clusters (5/121 conformations, data not shown), affirming FcαRI binding sensitivity to the structural arrangements of IgAs.

Holistically, the similarities and differences in the trends of IgAs across the VH families showed the importance of the systematic investigation of individual antibodies. It was shown here that generalizations of VH3 for superantigen bindings do not necessary apply across the board and that while IgA2s may play a smaller role in blood FcαRI immune cell activation than IgA1s, there were contributions by the V-regions that caused IgA2 to interact better with the receptor. In our analysis of FWR, VH1 was shown to be a possible VH-FWR for IgA1 and 2 grafting to retain antigen and FcαRI engagement even though VH3 remains a good candidate for humanization. On the other hand, our findings here agree with our previous investigations on other antibody isotypes (IgE) that the VH regions can affect FcR engagement and potentially play a role in disease pathogenesis with influence from superantigens. With the demonstration of the influence of PpL on IgA engagement of antigens and strong interactions between SpG and IgAs, the molecular mechanisms that underlie the interaction of normal flora at mucosal areas to mucosal antibodies IgA are better understood for future interventions.

## Materials and methods

### Recombinant protein production

All Trastuzumab and Pertuzumab VH and Vκ sequences used were described previously^49^. The genes were transformed into competent *E. coli* (DH5α) strains^79^ followed by plasmid extraction (Biobasic Pte Ltd) and sub-cloning into pTT5 vector (Youbio, China) using restriction enzyme sites, as previously performed^48–50,57,70,76^.

Transfection, production, and purification and were performed as described previously^49,57,80^.

### Binding affinity quantification

For Her2 binding, IgA variants were immobilized using PpL biosensor (Sartorius, Cat: 18–5185) or biotinylated anti-IgA antibody (Thermo Scientific, Cat: 7,102,882,500) bound on streptavidin biosensor (Sartorius, Cat: 18–5119) and subjected to free floating Her2 in solution to obtain the rate of association (ka), dissociation (kd), and equilibrium dissociation constant (KD). The program and steps used were as previously described^48–51,57,58,70,76^.

His-tagged FcαRI (Sino Biological, Cat: 10,414-H08H) were immobilized via Ni–NTA biosensor (Sartorius, Cat: 18–5101) and subject to free floating IgA variants to obtain the ka, kd, and KD. The program and steps used were as previously described^48–51,57,58,70,76^.

PpL (Sartorius, Cat: 18–5185), SpA (Sartorius, Cat: 18–5012), and SpG (Sartorius, Cat: 18–18-5083) biosensors were subject to free floating IgA variants to obtain the ka, kd and KD. The program and steps used were as previously described^48–51,57,58,70,76^.

## Supplementary Information

Supplementary Information.

## Data Availability

The datasets GENERATED/ANALYZED for this study is available upon request.
