# Supplementary material for: Variable-heavy (VH) families influencing IgA1&2 engagement to the antigen, FcαRI and superantigen proteins G, A, and L
Source: Sci Rep. 2022 Apr 20;12:6510. doi: 10.1038/s41598-022-10388-5 (PMC9020155; doi:10.1038/s41598-022-10388-5)
Supplement: Supplementary file 1 — Supplementary Information. [file 41598_2022_10388_MOESM1_ESM.docx]

Supplementary Material

# Supplementary Figures of Octet Loading


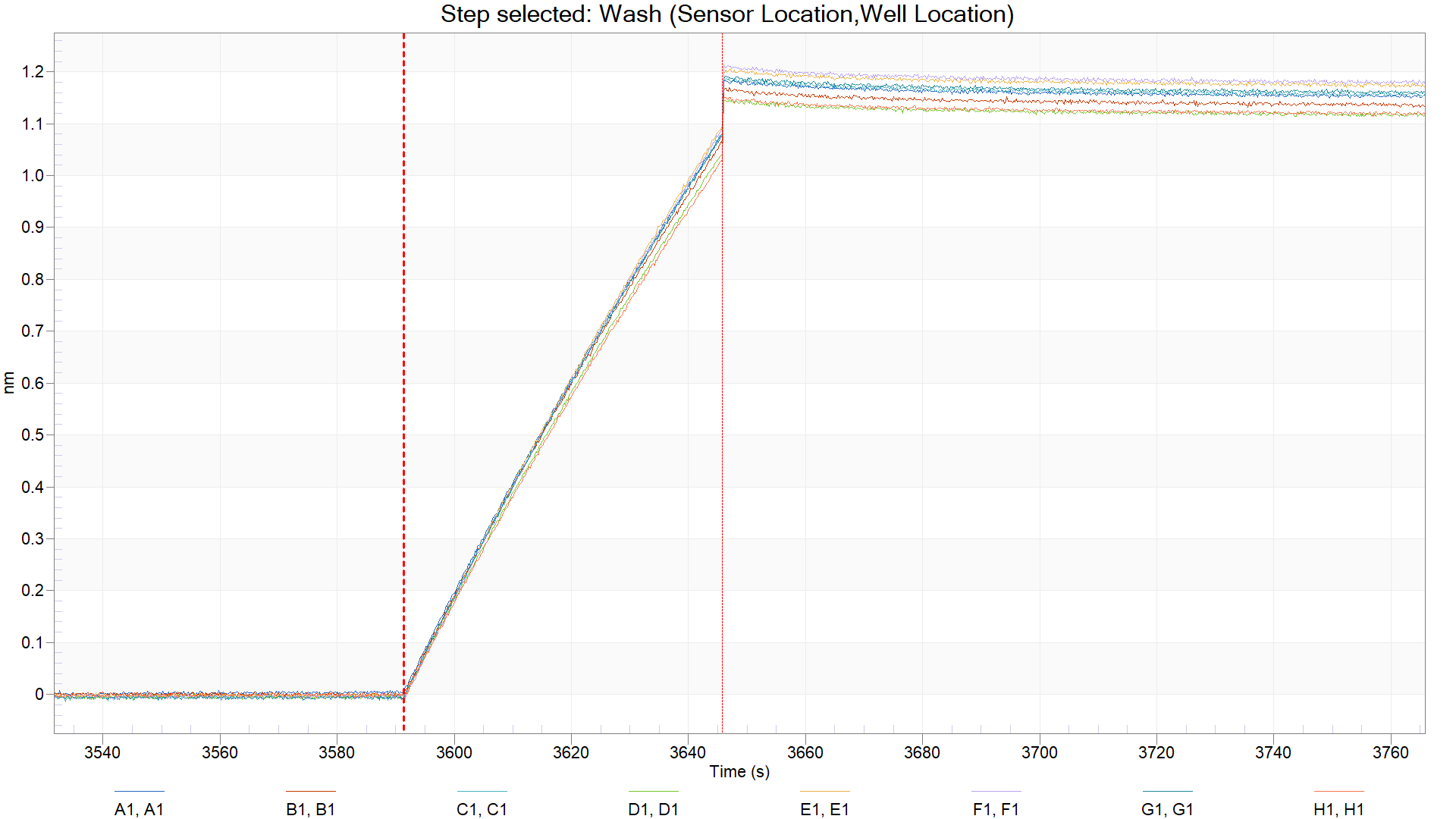


**Supplementary Figure S1:** Raw graph presentation of PVH4-IgA1 immobilizing on Protein L sensor (Red arrow).


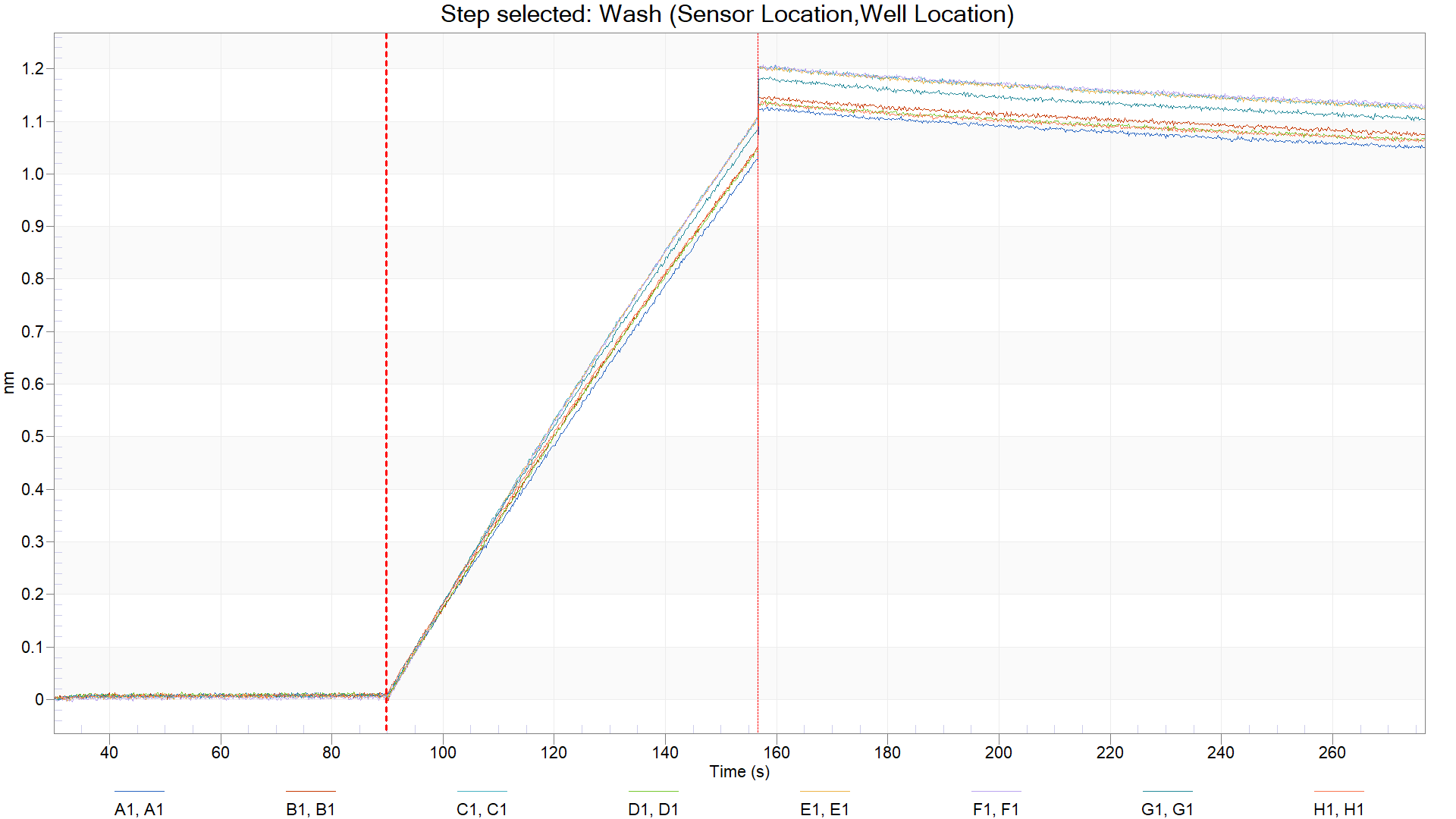


**Supplementary Figure S2:** Raw graph presentation of PVH2 IgA2 immobilizing on Protein L sensor (Red arrow).


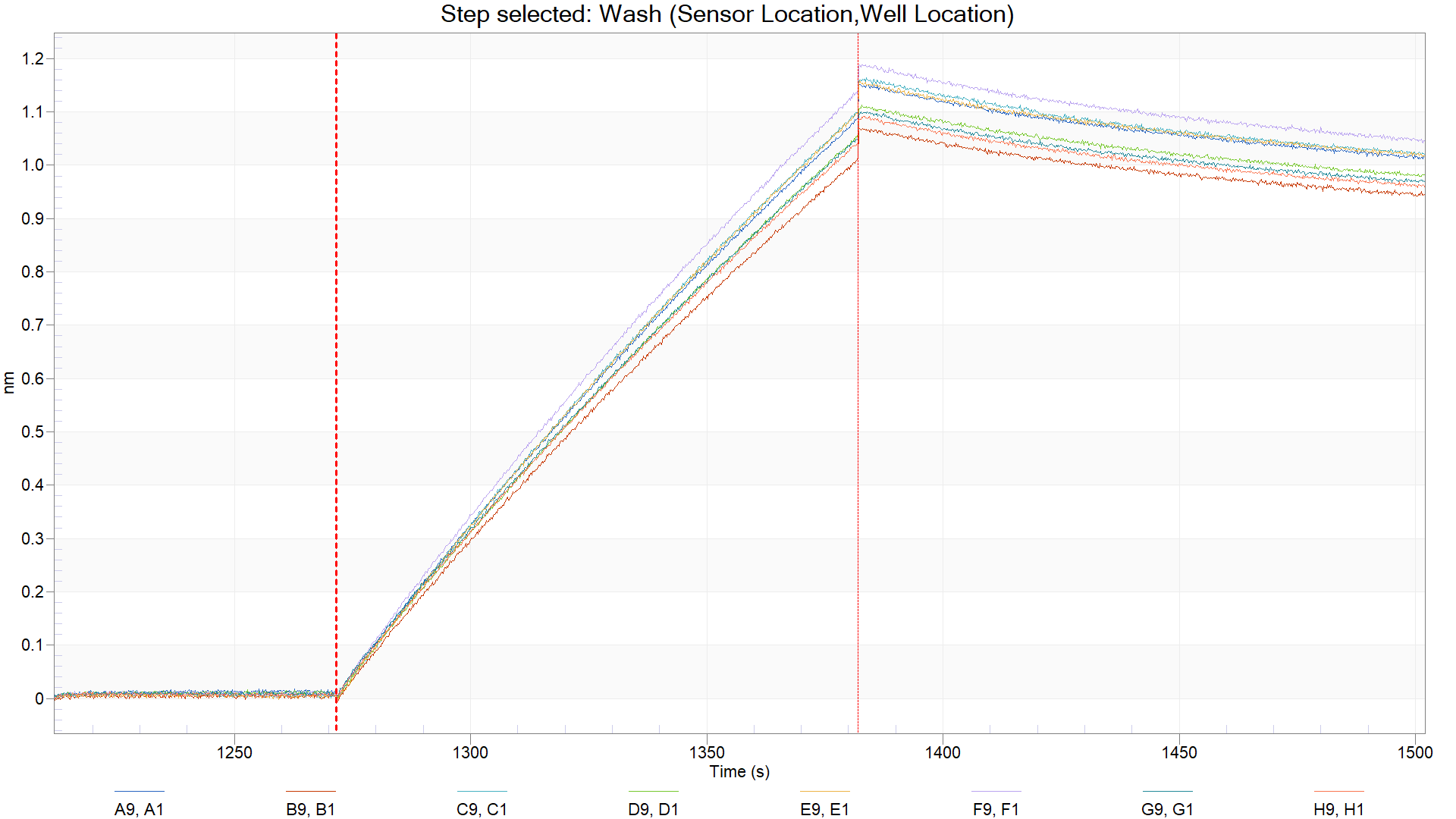


**Supplementary Figure S3:** Raw graph presentation of HVH4-IgA1 immobilizing on Protein L sensor (Red arrow).


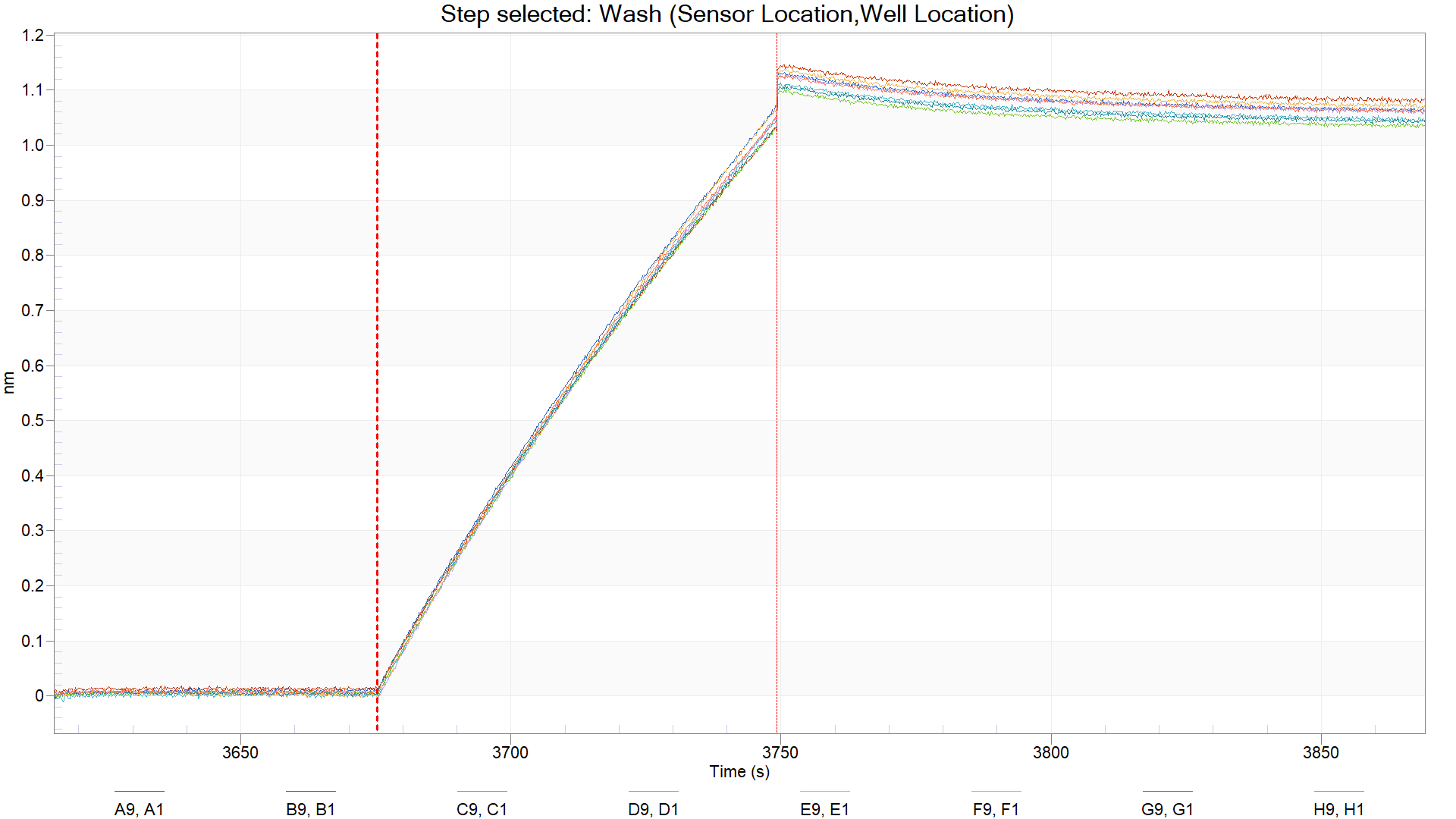


**Supplementary Figure S4:** Raw graph presentation of HVH7-IgA2 immobilizing on Protein L sensor (Red arrow).


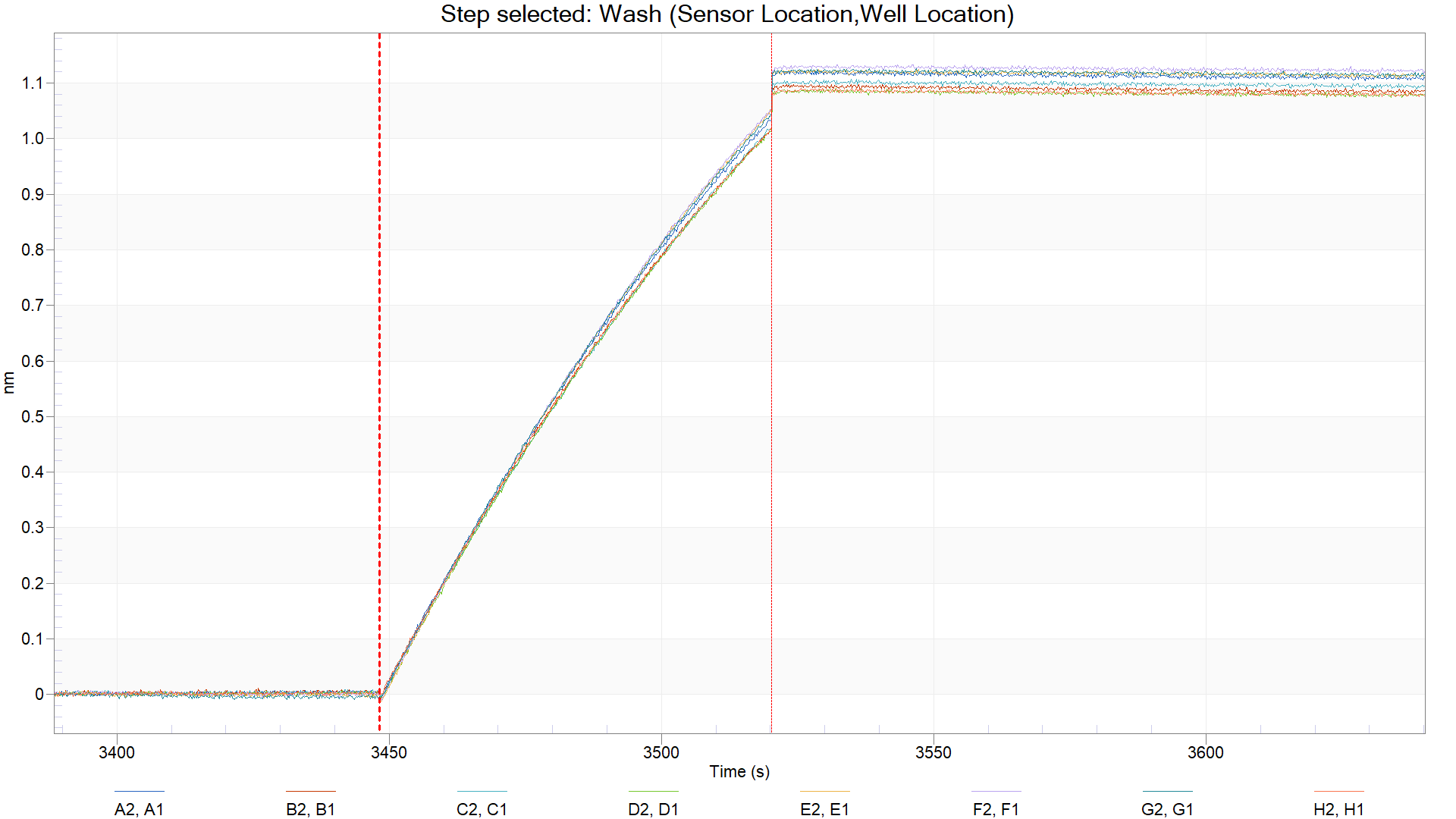


**Supplementary Figure S5:** Raw graph presentation of PVH3-IgA1 immobilizing on SA sensor coupled with biotinylated anti-IgA antibody (Red arrow).


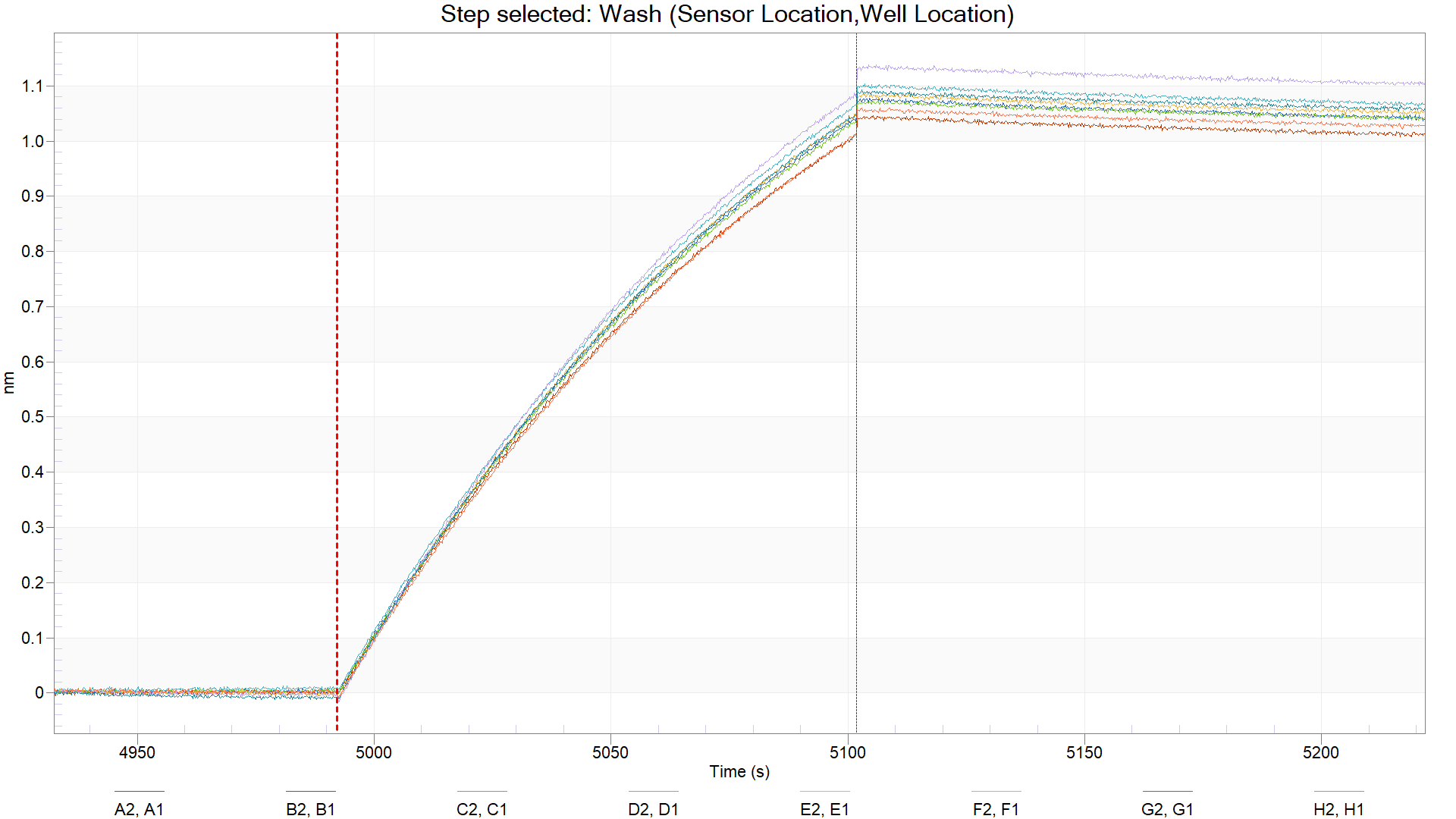


**Supplementary Figure S6:** Raw graph presentation of PVH5-IgA2 immobilizing on SA sensor coupled with biotinylated anti-IgA antibody (Red arrow).


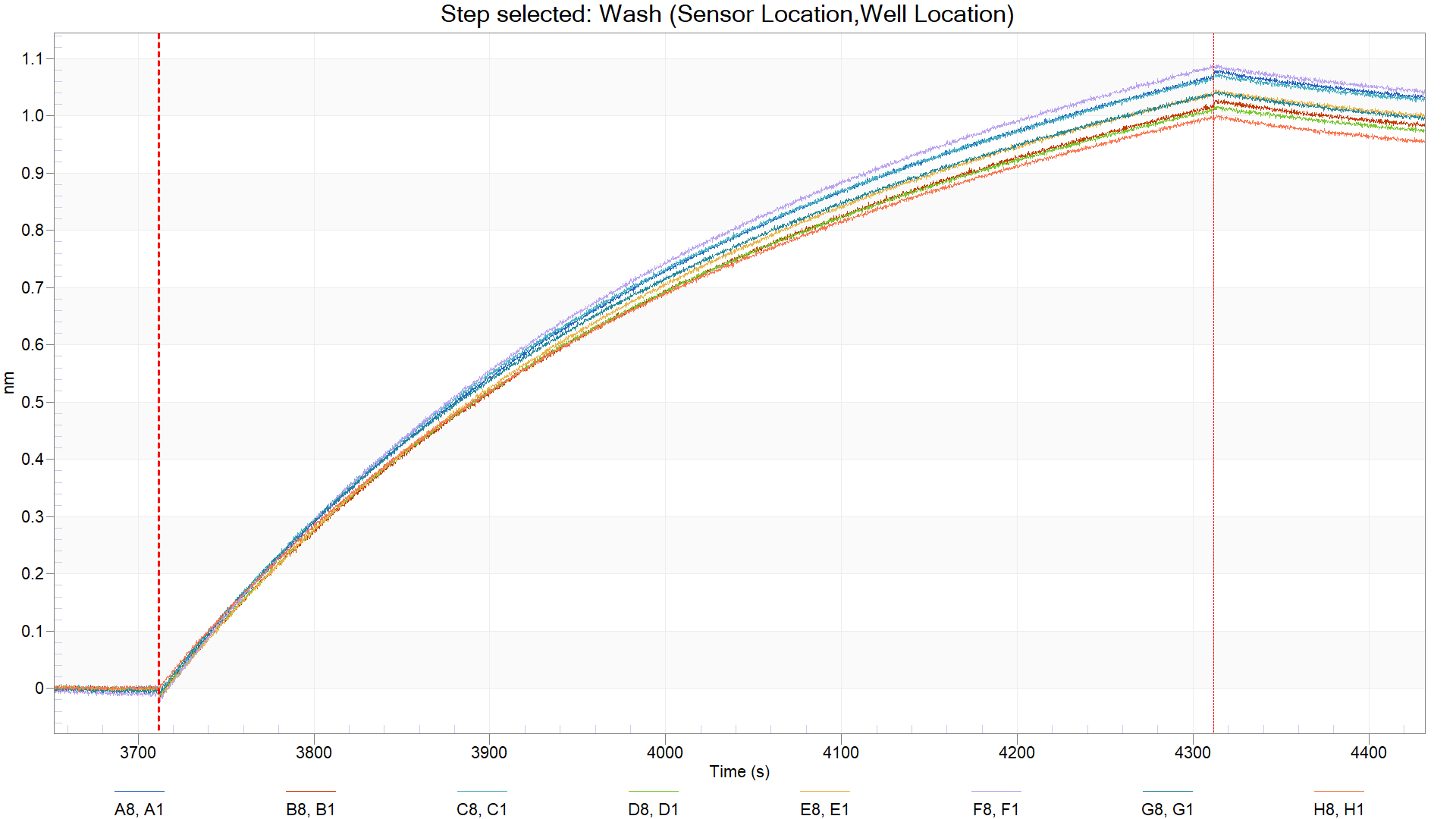


**Supplementary Figure S7:** Raw graph presentation of HVH1-IgA1 immobilizing on SA sensor coupled with biotinylated anti-IgA antibody (Red arrow).


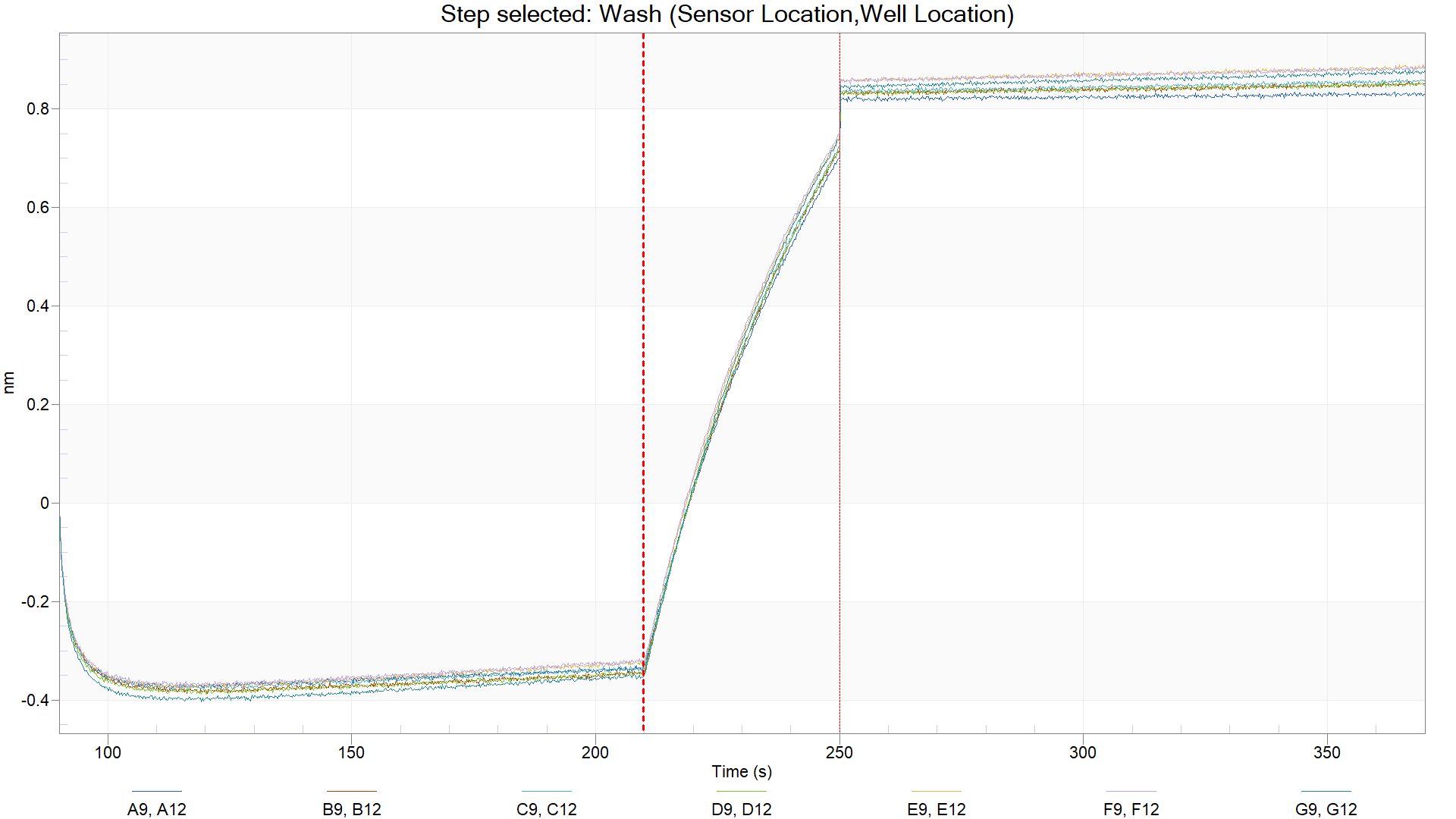


**Supplementary Figure S8:** Raw graph presentation of His-tagged FcαRI immobilizing on NTA sensor binding to PVH1-IgA1 (Red arrow).


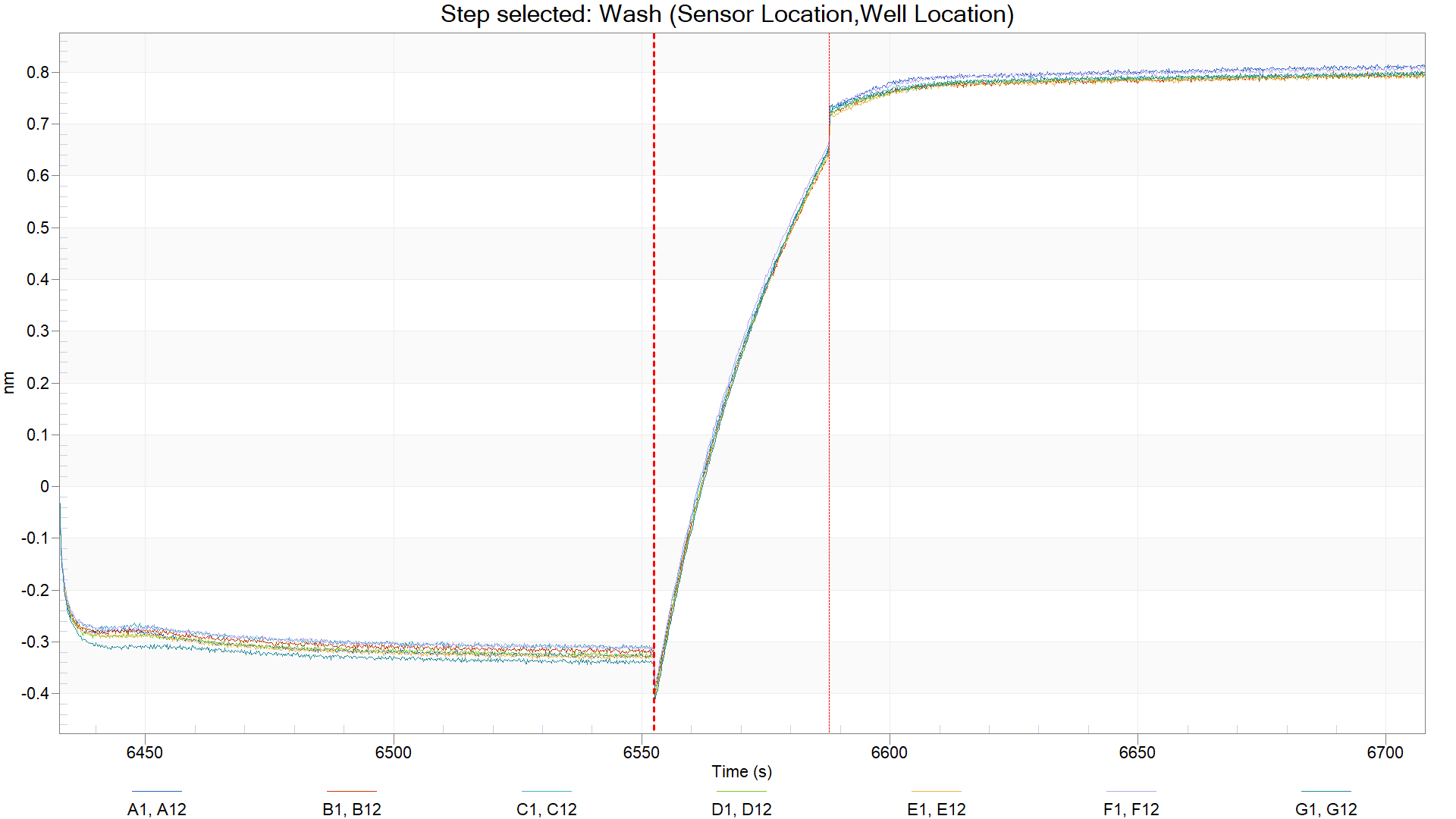


**Supplementary Figure S9:** Raw graph presentation of His-tagged FcαRI immobilizing on NTA sensor binding to PVH6-IgA2 (Red arrow).


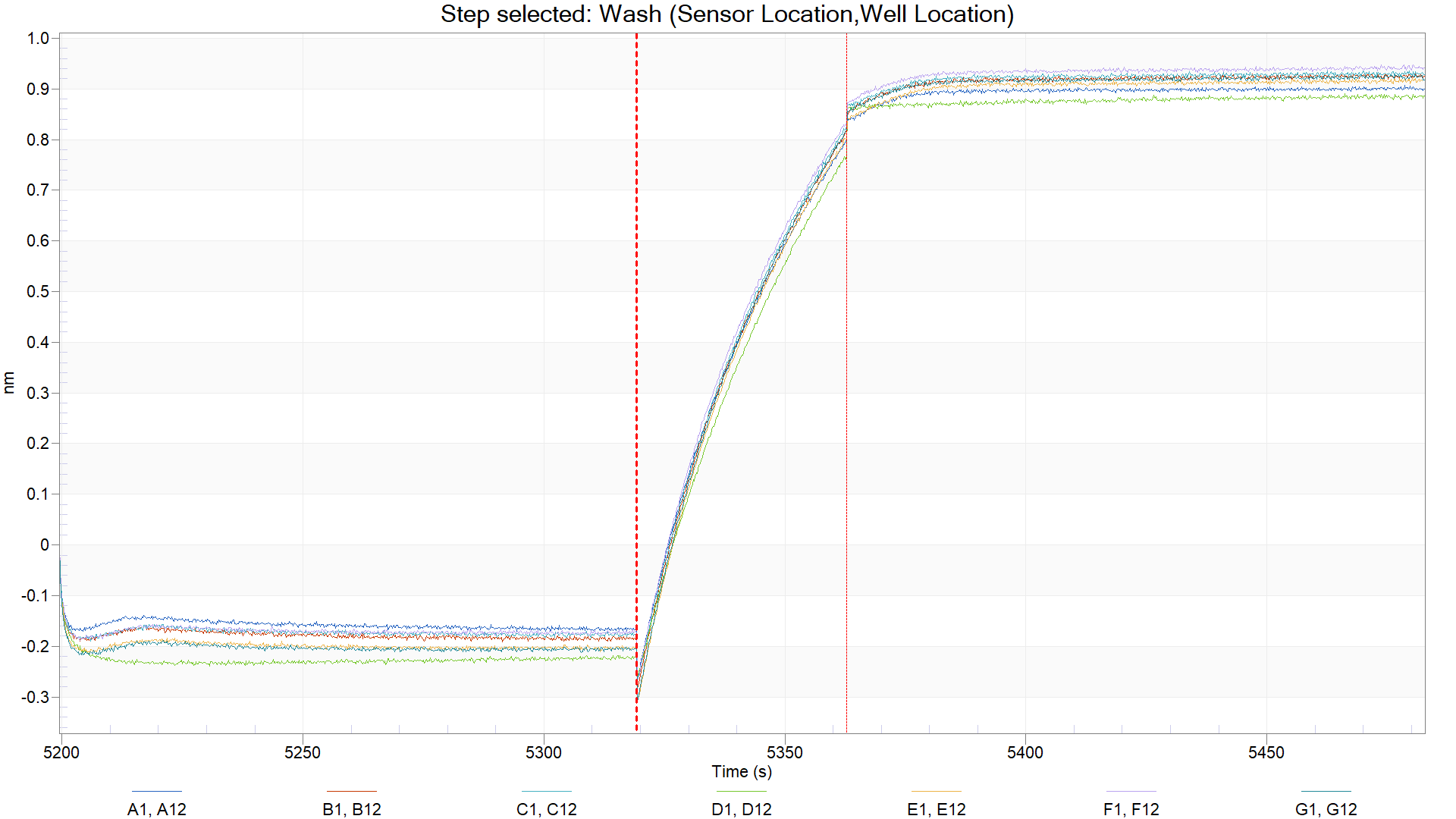


**Supplementary Figure S10:** Raw graph presentation of His-tagged FcαRI immobilizing on NTA sensor binding to HVH5-IgA1 (Red arrow).


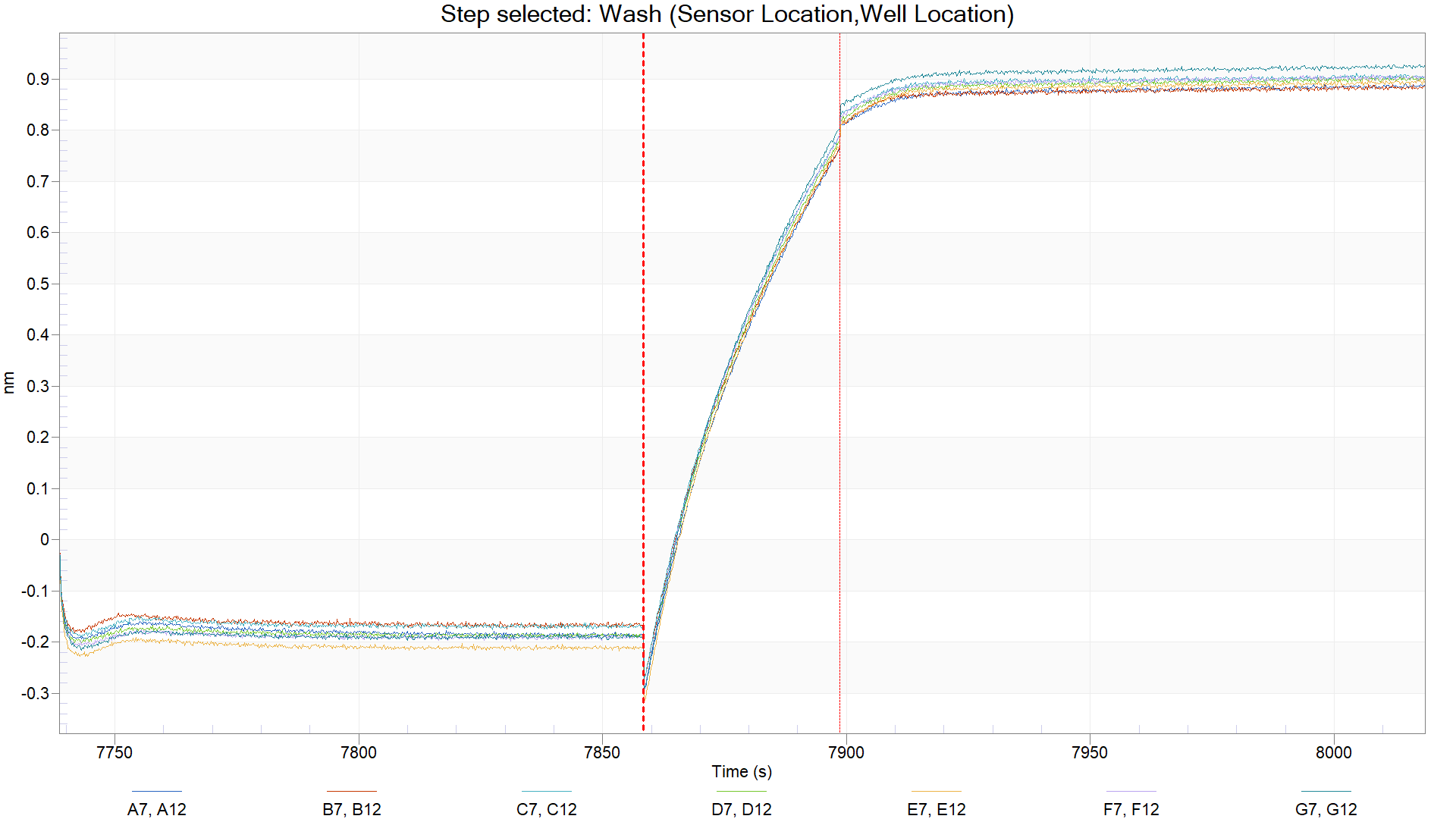


**Supplementary Figure S11:** Raw graph presentation of His-tagged FcαRI immobilizing on NTA sensor binding to HVH7-IgA2 (Red arrow).

**Octet loading exceptions with poor loading**


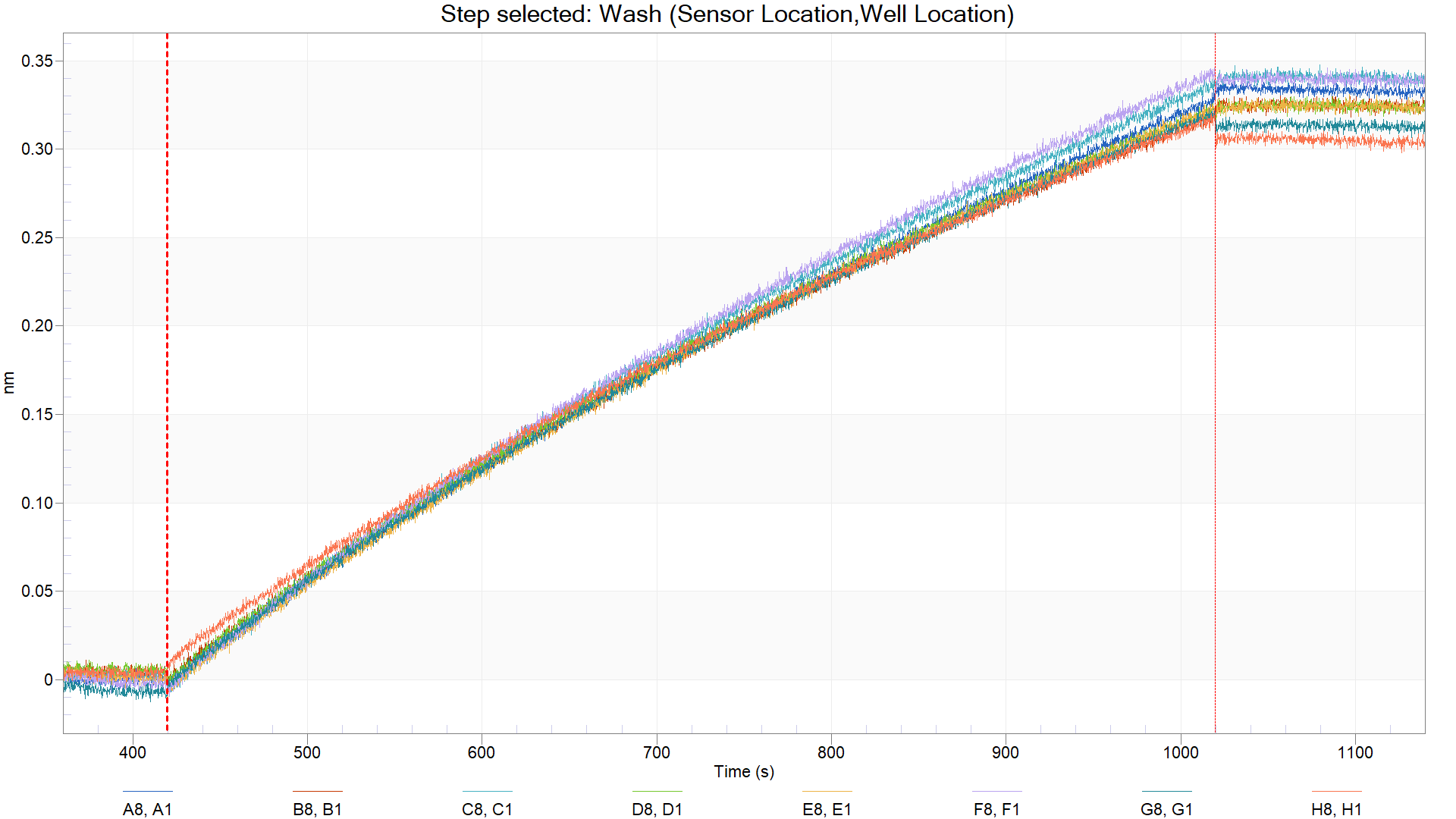


**Supplementary Figure S12:** Raw graph presentation of HVH4-IgA2 immobilizing on SA sensor coupled with biotinylated anti-IgA antibody (Red arrow).

# Computational Structural Analysis of the IgAs

Modeling of full length IgA1 and IgA2 Pertuzumab and Trastuzumab VH2, VH3, VH4 and VH5 variants

The Fv (pairing VHx-Vκ1) models of the Pertuzumab and Trastuzumab VH2, VH3, VH4, and VH5 variants were first constructed using the online ROSIE Antibody protocol ^1^ . For those modelling jobs reported as “failed” by ROSIE (i.e. Trastuzumab VH2 and VH4 variants) due to the server overload, the standalone Rosetta package ^2^ was used for re-grafting.

We first tested the performance consistency between the two versions by comparing the CDRs modelling results of a few variants e.g., Pertuzumab VH1, VH2, VH4, and VH5 (data not shown). It was observed that the results from both ROSIE and Rosetta standalone versions demonstrated the consistency of the computational CDRs grafting and modelling, i.e., results of fluctuations (RMS) and solvent exposures of the top-100 ranked modelled CDRs in both the versions were highly correlated (with calculated Pearson correlation R=0.87, p-value < 10-4). Hence, we proceeded to use the complementary results from the Rosetta standalone version. As the standalone version required more manual interventions, with exception of the Trastuzumab VH2 and VH4 variants, we used the ROSIE results for the other variant models.

Subsequently, the lowest scored grafted Fv model of each Pertuzumab/Trastuzumab VHx variant was selected for computational joining onto the heavy chain Cα1-Cα3 backbone of the IgA1 and IgA2, previously constructed ^3^. Cκ was used for the light chain constant region.

*N*-linked glycans (referenced from PDB: 1OW0) were attached to several asparagine residues of the full-length IgAs models, i.e., at N263 and N459 of the IgA1. Since IgA2 contains two additional conserved N-glycan sites ^4^, the *N*-linked glycans were attached at N166 and N324 together with N251 and N446 of the IgA2 models. In addition, *O*-linked glycans (α2,3-linked to Gal and α2,6-linked to GalNAc) were attached to serine/threonine of the proline-rich hinge of the IgA1 at T228, S230, S232, T233, T236, and S240 ^4,5^. The attachment of glycans were performed using CHARMM GUI *Glycans Reader & Modeler* ^6^. All the full-length glycan-attached IgAs models were energy-minimized using GROMACS v2019 ^7^.

Molecular docking of IgA Pertuzumab and Trastuzumab VH2, VH3, VH4, and VH5 variants to FcαRI

The FcαRI domain was first extracted from the crystal structure of the human FcαRI-bound IgA1-Fc (PDB: 1OW0) and independently docked to the C-region (chain A) of each IgA1/IgA2 Pertuzumab and Trastuzumab VH2, VH3, VH4, and VH5 models using HADDOCK 2.4 server ^8^ with default settings. The binding regions (active residues) on IgA1 (IgA2) are L256 (L243), L257 (L244), L258 (L245), R382 (R369), S387 (S374), E389 (E376), M433 (M420), E437 (E424), L439 (L426), L441 (L428), A442 (A429), F443 (F430), Q445 (Q432) and on FcαRI are R52, R53, L54, K55, F56, Y35, G84, H85, R82. These residues were derived from work by Herr *et al ^9,10^*.

Since the HADDOCK server accepts only standard residues for protein-protein docking, all the glycans were removed prior to the docking. However, the original positions of all glycans were later used to assess the resulting docked complex conformations.

Only docked FcαRI-IgAs complexes that satisfied the following criteria were selected for further analyses: (i) there is no overlap between the resulting FcαRI conformation and the modeled glycans, (ii) the resulting FcαRI bound conformation reflects the central hydrophobic cores of L258, L441, M433, and F443 (of the IgAs-Fc) recruiting the packing residues Y35, F56, and H85 of the FcαRI, as previously reported ^10^. When more than one docked cluster conformations were satisfied with the criteria above, the cluster with better HADDOCK score (higher rank) was selected and its top three conformations were used as replicates for further analyses. The contacts at the FcαRI-Fcα interfaces of the resulting docked complexes were then determined using CIPS ^11^.


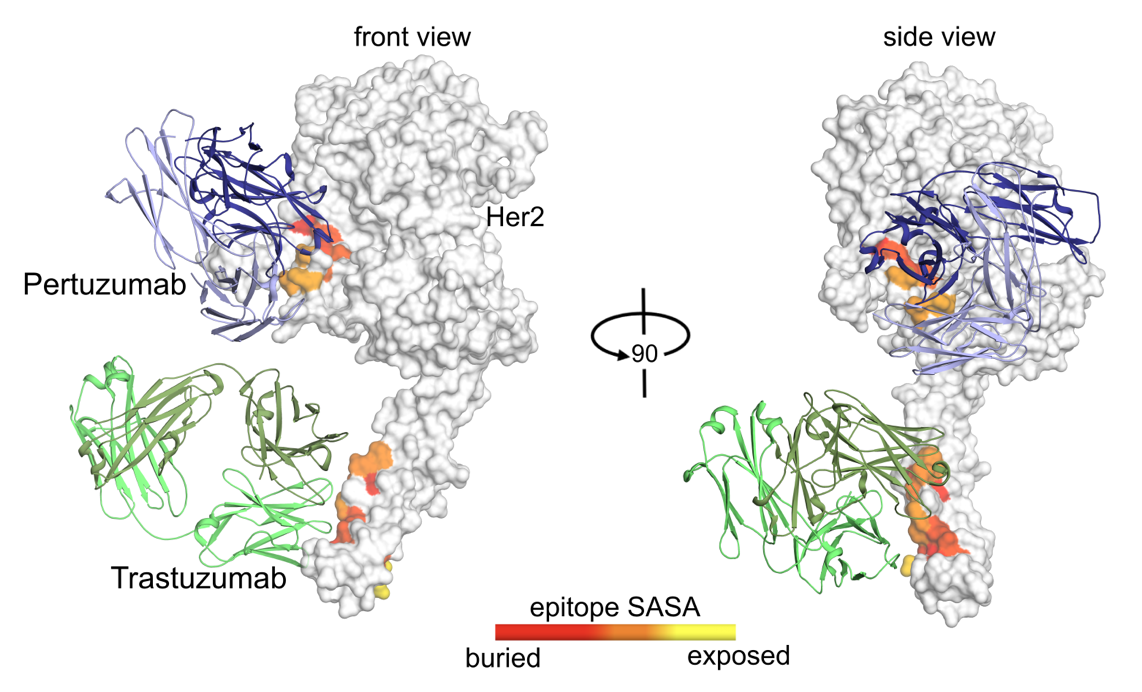


**Supplementary Figure S13:** Different Her2 epitopes with respect to Trastuzumab (dark and light green for heavy and light chain, respectively) and Pertuzumab (dark and light blue) and their exposures quantified using Solvent Accessible Surface Are (SASA).

**Differential FcαRI binding between Trastuzumab and Pertuzumab IgA1 variants**


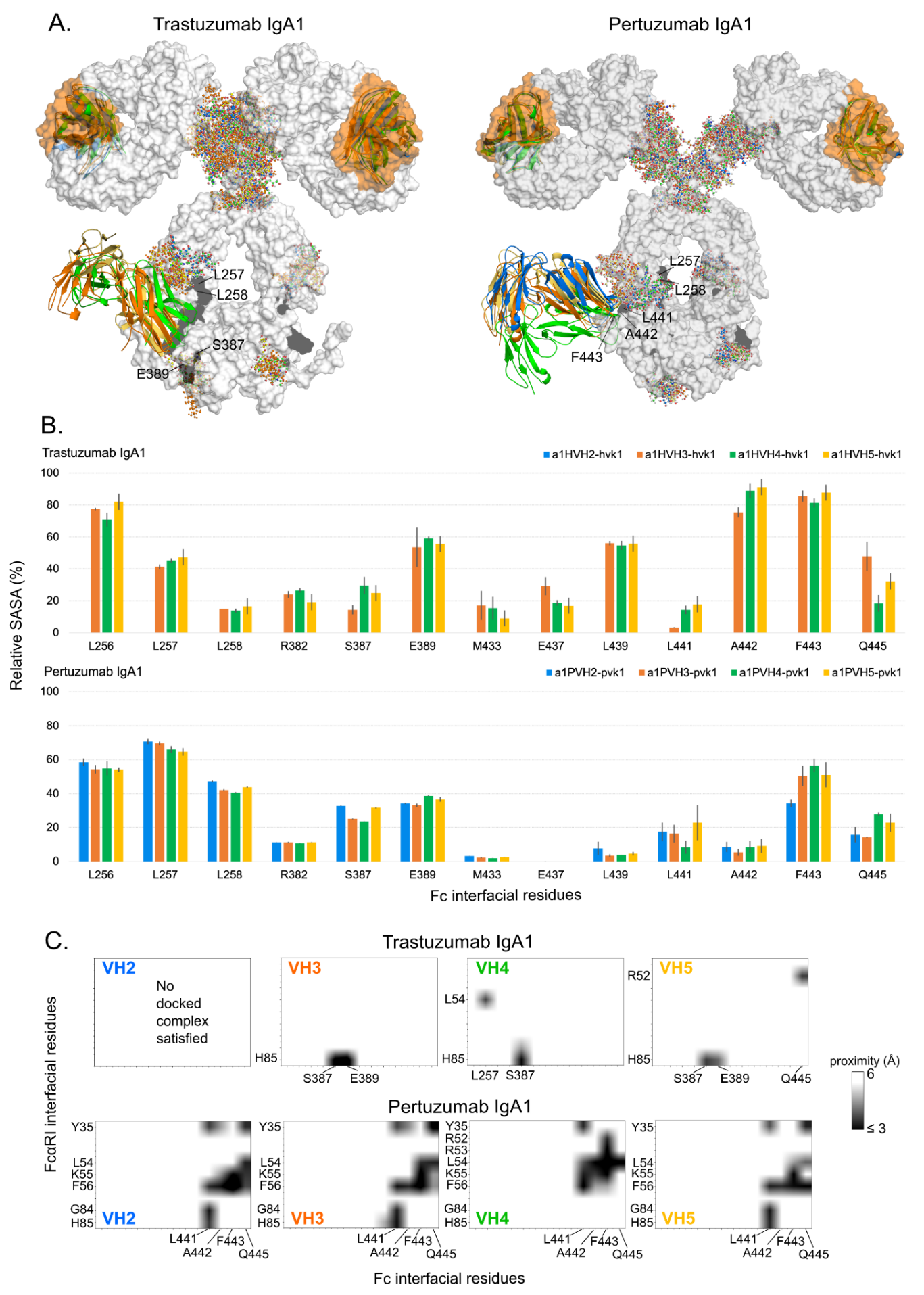


**Supplementary Figure S14**: Computational analysis of FcαRI binding by Trastuzumab/Pertuzumab IgA1 variants. (A). Docked complexes of FcαRI (shown in colored ribbon) to Trastuzumab (VH3, VH4, and VH5) and Pertuzumab (VH2, VH3, VH4, and VH5) IgA1 variants. The symmetrical active sites on the Fc regions are filled in black, with key active residues involved in FcαRI contacts labelled. For simplification, only the backbone of the Trastuzumab/Pertuzumab VH3 variants are presented in white surface, whereas the different Fv and the resulting FcαRI binding modes are colored accordingly to the VH2 (blue), VH3 (orange), VH4 (green), and VH5 (yellow) families. The attached glycans are shown in balls and sticks with atoms following the color scheme as: carbon (accordingly with those of the VH families), oxygen (red), hydrogen (white). (B, C). Interaction analyses at the FcαRI-Fcα interfaces with relative solvent accessibility surface area (SASA) of the active site residues on the IgA1-Fc regions (B) and distance matrices between the interfacial residues (C). The color scheme are as in (A). The visualizations were generated using PyMOL v.2.3.2 ^12^.

Distinct FcαRI binding kinetics measurements were observed for different IgAs VH variants (e.g., the VH2 and VH4 in comparison to VH3/VH5 in both Trastuzumab and Pertuzumab variants as shown in Table 2) indicating the distal effects between the V- and FcαRI binding site at the C-regions, in agreement with our previous findings ^3^.

In contrast to the limited motion of the truncated IgA1 Fc alone (using PDB: 1OW0), the C-region of full-length IgA1 exhibited larger dynamics motions with respect to the long and rigid proline-rich hinge (Supplementary Figure S16). The increased mobility was less pronounced for IgA2 with reduced swing-like intradomain motions as observed around the IgA1 Cα2-Cα3 joint (surrounding the FcαRI binding sites). The signal was propagated via the hinge connected by two disulfide bridges. This suggests that this varying mobility resulted in various exposures of the FcαRI-binding sites on the C-region with respect to IgA1 or IgA2.

Among the Pertuzumab and Trastuzumab IgAs, FcαRI binding kinetics measurements of the Trastuzumab VH2 and VH4 variants were distinct from the other VH families and were beyond the measurement limits. To characterize the structural FcαRI binding in these variants, we first generated FcαRI-bound IgA1 Pertuzumab and Trastuzumab variants of VH2, VH3 (as control), VH4, and VH5 (Supplementary Figure S14A), using the HADDOCK 2.4 server ^8^.

We performed the docking analysis first for the IgA1 variants because the long-range communications between the V- and C-regions were the most pronounced among the full IgA1, IgA2 and the truncated Fc due to the hinge variations (Supplementary Figures S16), and the referenced experimental structural complex information of the FcαRI binding modes is currently available only for IgA1, e.g., PDB: 1OW0.

Apart from the Trastuzumab IgA1 VH2 models (which resulted in docked complexes with FcαRI-bound configurations overlapping with the modeled glycans), the reasonable FcαRI-bound conformations to the other IgA1 models of VH2, VH3, VH4, and VH5 diverged into two different binding modes at around the FcαRI-binding region on the IgA1-Fc with respect to Trastuzumab or Pertuzumab variants (Supplementary Figure S14A). As observed in both the Trastuzumab and Pertuzumab IgA1 models, the *N*-linked glycans attached to N263 of the Cα2 domain shielded the two hydrophobic L257 and L258 residues (more pronounced in the Pertuzumab IgA1 models) that were among the reported FcαRI-binding residues in the truncated IgA1-Fc ^10^, i.e. 1OW0. These occupations of the *N*-linked glycans likely had different accommodation of the FcαRI binding to the C-region between the Trastuzumab and Pertuzumab variants. In addition, the differences of the FcαRI binding orientations in these IgA1 models as compared to the referenced FcαRI-Fcα complex (1OW0) were likely due to the lack of the C-terminal tail of the Fc domain in the truncated Fc of the referenced complex 1OW0.

Since hydrophobic packing was reported as a crucial factor in the FcαRI binding to IgA1 ^9^, solvent accessibility surface area (SASA) was quantified to determine the contact interfaces of these docked complexes ( Supplementary Figure S14B). It was shown that the hydrophobic packing core at the central IgA1 Fc were maintained with SASA<30% burying M433 and L441, and deeper buried L439 and A442 in Pertuzumab bound complexes. Distance matrices at the FcαRI-Fcα interfaces to determine the proximity of these interfacial residues (Supplementary Figure S14C) revealed two distinct interfacial contact tendencies between those Trastuzumab and Pertuzumab IgA1 variants.

In the docked complexes of the Trastuzumab IgA1 VH3, VH4, and VH5 variants, the FcαRI interacted mostly with the Fcα polar residues S387 and/or E389. Additional interactions with E389 via electrostatics with H85 (of FcαRI) in the VH3 and VH5 variants may have contributed to stronger FcαRI bindings as compared to those in the VH4, in agreement to the experimental binding kinetics measurements shown in Table 2. The essential contribution of E389 into the FcαRI binding was also reported in a previous mutagenesis study ^9^.

For the Pertuzumab IgA1 variants, signature hydrophobic interactions were found at the FcαRI-Fcα interfaces of the VH2, VH3, VH4, and VH5 variants (Supplementary Figure S14 C, bottom panels) such as the hydrophobic interactions of L441 (of Fcα) and Y35, F56 and/or H85 (of FcαRI) and of F443 (Fcα) against the hydrophobic core of Y35, L54, F56 and the aliphatic K55 of the FcαRI.

The binding contribution of the two residues L257 and L258 to the FcαRI were not as clearly exhibited in these Trastuzumab/Pertuzumab IgA1 VH2, VH3, VH4, and VH5 variants as compared to those in the referenced truncated Fc complexed with the FcαRI.

**Superficial binding of the FcαRI to the IgA2 C-regions**


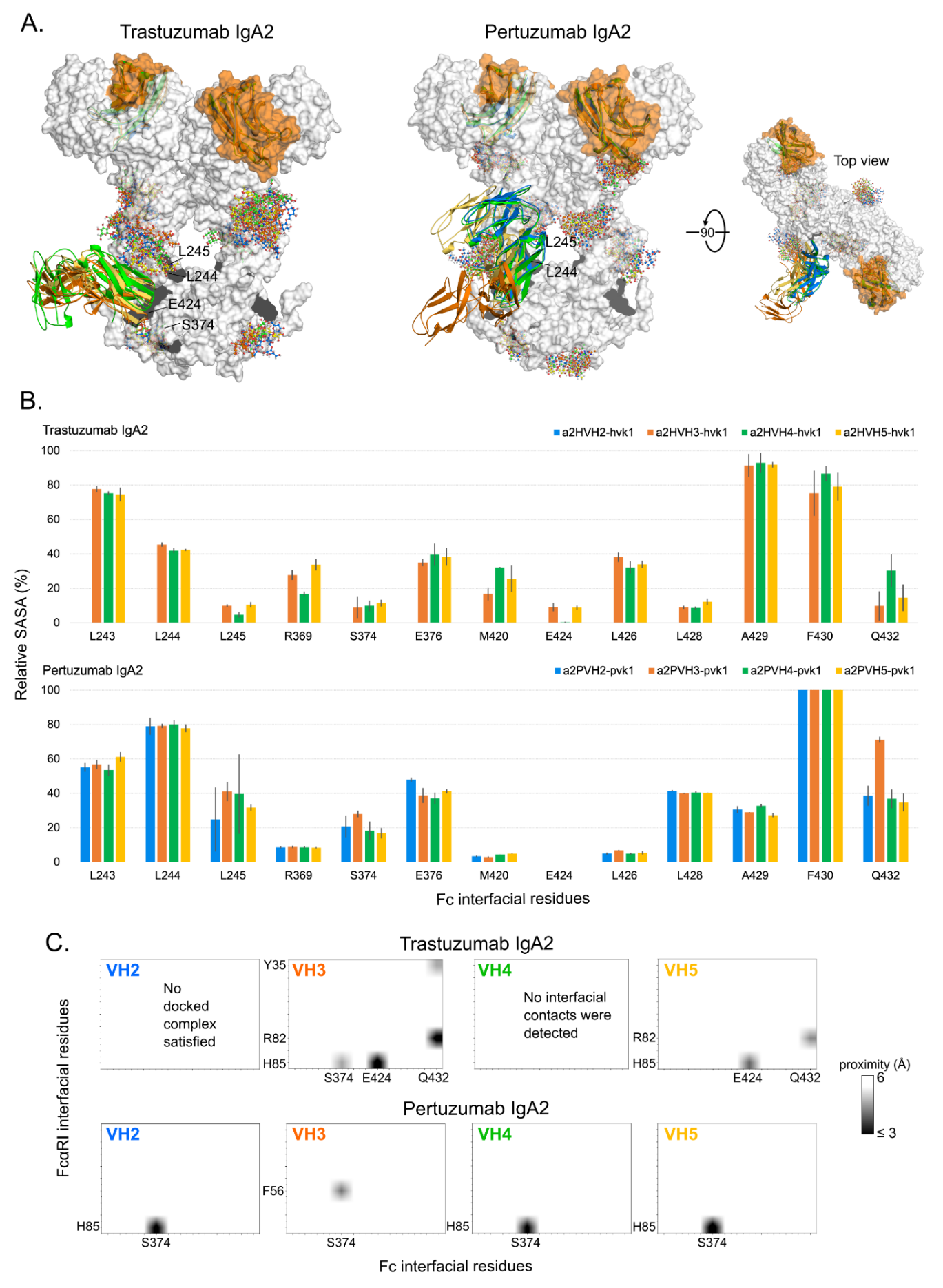


**Supplementary Figure S15**. Computational analysis of FcαRI binding by Trastuzumab/Pertuzumab IgA2 variants. (A). Docked complexes of FcαRI (shown in colored ribbon) to Trastuzumab (VH3, VH4, and VH5) and Pertuzumab (VH2, VH3, VH4, and VH5) IgA2 variants. The symmetrical active sites on the Fc regions are filled in black, with key active residues involved in FcαRI contacts labelled. For simplification, only the backbone of the Trastuzumab/Pertuzumab VH3 variants are presented in white surface, whereas the different Fv and the resulting FcαRI binding modes are colored accordingly to the VH2 (blue), VH3 (orange), VH4 (green), and VH5 (yellow) families. The attached glycans are shown in balls and sticks with atoms following the color scheme as: carbon (accordingly with those of the VH families), oxygen (red), hydrogen (white). (B, C). Interaction analyses at the FcαRI-Fcα interfaces with relative solvent accessibility surface area (SASA) of the active site residues on the IgA2-Fc regions (B) and distance matrices between the interfacial residues (C). The color scheme are as in (A).

The lack of experimentally determined structures of the FcαRI-IgA2 complexes moved us to computationally investigate the FcαRI binding mechanism in the IgA2 variants by docking the FcαRI to the C-regions of the Trastuzumab/Pertuzumab IgA2 VH2, VH3, VH4, and VH5 models. We performed the docking with similar references from those of the IgA1 dockings with the highly identical sequences of the IgA1 and IgA2 Fc domains (Cα2-Cα3).

Around the two additional *N*-linked glycan sites on the IgA2 C-region, i.e. N166 (Cα1) and N324 (Cα2) ^4^, the *N*-linked glycans were found to be more densely distributed around the C-regions, particularly at the Cα1-Cα2 joint when compared to the IgA1 models. This suggests higher structural constraints in the whole IgA2 mobility, thereby influencing the FcαRI-binding region on the IgA2 C-region.

Apart from the Trastuzumab IgA2 VH2 variant, the other Trastuzumab/Pertuzumab IgA2 VH2, VH3, VH4, and VH5 variants formed complexes with the FcαRI (Supplementary Figure S15A) based on the selection criteria onto the resulting docked FcαRI-IgA2 complexes (see supplementary methods above).

Within the Trastuzumab IgA2 complexes, the VH3 and VH5 docked complexes shared similar FcαRI binding modes and interfacial SASA profiles (orange and yellow in Supplementary Figure S15A-B) in contrast to the VH4 variants. Distance matrices at the FcαRI-Fcα interfaces revealed no contacts among the interfacial residues in the Trastuzumab VH4 complexes (Supplementary Figure S15C).

Within the Pertuzumab IgA2 variants, the FcαRI formed a distinct binding orientation in the VH3 docked complexes as compared to those of VH2, VH4, and VH5. In the FcαRI-Fcα interfaces of these Pertuzumab IgA2 variant complexes, most of the contacts involved the polar residues S374 (corresponding to S387 in the IgA1) with the H85 of the FcαRI except for Pertuzumab IgA2 VH3 complex, which had the S374 residue detected more distant (~4.3Å) from FcαRI F56 (Supplementary Figure S15C). The interfacial SASA profiles of these Pertuzumab IgA2 variants (Supplementary Figure S15B) showed that the hydrophobic core of the IgA2 C-region (involving M420, L428, A429) remained relatively intact (e.g., SASA<40%), similar to those in the Pertuzumab IgA1 variants (corresponding to M433, L441, A442, shown in Supplementary Figure S14B). None of these hydrophobic residues were detected in the FcαRI-Fcα interfaces of the Pertuzumab IgA2 docked complexes, suggesting superficial contacts of the FcαRI at the binding surface of the Pertuzumab IgA2 variants, particularly for the VH3 variant (e.g., Supplementary Figure S15A-right panel, top view) when compared to the Pertuzumab IgA1 variant complexes.

**
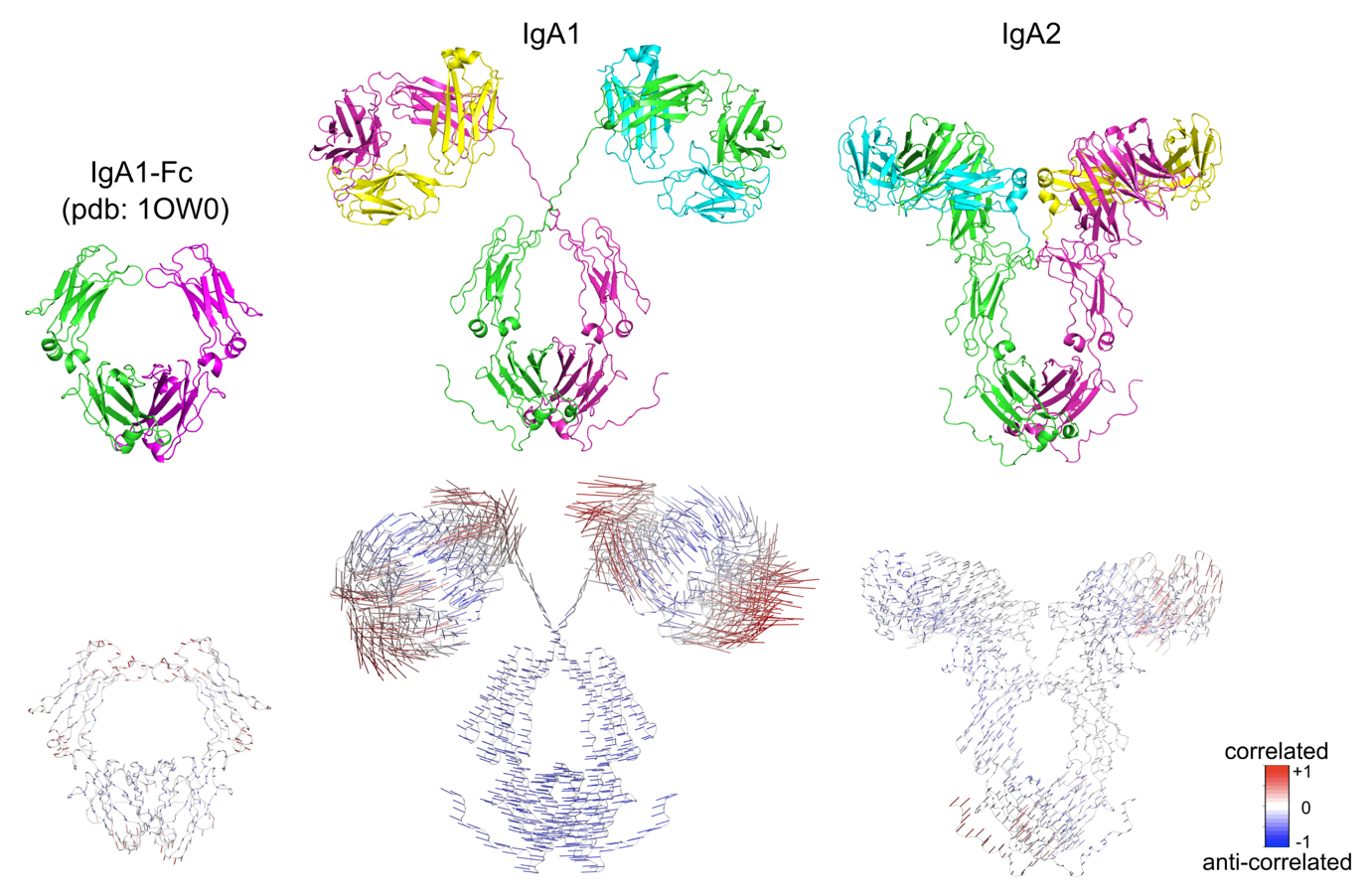
**

**Supplementary Figure S16:** Comparison of correlated domain motions of the full-length IgA1 and IgA2. Directed motions (shown as arrows) of the domains and the motion magnitude (shown by the arrow length) indicate the long-range effects between the V- and C-regions in the full-length antibody variants that resulted in increased motion magnitudes of the C-region as compared to the truncated IgA1 C-region (PDB: 1OW0). The effect is less pronounced in the IgA2s due to the differences of hinge length. The Bio3D package ^13^ was used to perform normal mode analysis on the unbound IgA1 and IgA2 Trastuzumab VH3 models and the IgA1-Fc alone (1OW0). Fluctuations were presented using the first non-trivial modes, i.e., modes 7, for each model. The visualization was performed using PyMOL v.2.3.2.

# Supplementary Tables

Supplementary Table S1. BLI measurements of PpL and anti-IgA biosensor immobilized Pertuzumab (PVH) and Trastuzumab (HVH) 1-7 IgA1 and IgA2 interaction to Her2. The KD, ka and kd values with the standard error of each variant are shown accordingly. The KD differences between the two immobilization methods were shown in the last column with “x” indicating the fold differences. Poor response (PR) indicates that the antibody construct did not yield reliable ka and kd measurements (in triplicates) and NIL indicates ‘not comparable’ due to the lack of data (PR) under Protein L immobilization. All measurements were performed in triplicates and rounded off to 2 decimal places.

| Binding measurement to Her2 | | | | | | | | | |
| --- | --- | --- | --- | --- | --- | --- | --- | --- | --- |
| Construct | Protein L Immobilization | | | Anti-IgA Immobilization | | | Immobilization KD Differences | | |
|  | KD (10^-9^) | ka (10^4^) | kd (10^-4^) | KD (10^-9^) | ka (10^4^) | kd (10^-4^) | KD | ka | kd |
| PVH1-IgA1 | 7.53 ± 0.03 | 11.74 ± 0.05 | 8.74 ± 0.02 | 0.23 ± 0.01 | 32.90 ± 0.14 | 0.78 ± 0.02 | ~32.7x | ~2.8x | ~11.2x |
| PVH2-IgA1 | 13.43 ± 0.47 | 15.86 ± 0.52 | 21.53 ± 0.27 | 2.90 ± 0.06 | 32.09 ± 0.54 | 9.28 ± 0.09 | ~4.6x | ~2.0x | ~2.3x |
| PVH3-IgA1 | 9.86 ± 0.05 | 10.35 ± 0.04 | 10.20 ± 0.03 | 0.68 ± 0.01 | 33.18 ± 0.15 | 2.26 ± 0.02 | ~14.5x | ~3.2x | ~4.5x |
| PVH4-IgA1 | 8.95 ± 0.04 | 8.05 ± 0.03 | 7.12 ± 0.02 | 0.58 ± 0.01 | 23.62 ± 0.12 | 1.38 ± 0.02 | ~15.4x | ~2.9x | ~5.1x |
| PVH5-IgA1 | 50.55 ± 0.81 | 9.17 ± 0.14 | 46.24 ± 0.22 | 7.09 ± 0.11 | 27.57 ± 0.39 | 19.53 ± 0.10 | ~7.1x | ~3.0x | ~2.3x |
| PVH6-IgA1 | 36.62 0.16 | 3.88 ± 0.01 | 13.76 ± 0.02 | 6.57 ± 0.04 | 11.55 ± 0.05 | 7.58 ± 0.02 | ~5.5x | ~2.9x | ~1.8x |
| PVH7-IgA1 | 17.60 ± 0.07 | 8.02 ± 0.03 | 13.97 ± 0.02 | 2.67 ± 0.02 | 24.33 ± 0.11 | 6.51 ± 0.02 | ~6.5x | ~2.9x | ~2.1x |
| PVH1-IgA2 | 7.64 ± 0.05 | 14.70 ± 0.09 | 11.10 ± 0.04 | 0.47 ± 0.01 | 29.75 ± 0.17 | 1.41 ± 0.03 | ~16.2x | ~2.0x | ~7.8x |
| PVH2-IgA2 | 10.37 ± 0.29 | 14.13 ± 0.35 | 14.69 ± 0.18 | 3.45 ± 0.06 | 27.10 ± 0.39 | 9.33 ± 0.08 | ~3.0x | ~1.9x | ~1.5x |
| PVH3-IgA2 | 8.40 ± 0.05 | 11.42 ± 0.06 | 9.56 ± 0.03 | 0.85 ± 0.01 | 28.81 ± 0.17 | 2.46 ± 0.03 | ~9.8x | ~2.5x | ~3.8x |
| PVH4-IgA2 | 7.25 ± 0.04 | 8.26 ± 0.04 | 5.99 ± 0.02 | 0.95 ± 0.01 | 20.84 ± 0.12 | 1.99 ± 0.03 | ~7.6x | ~2.5x | ~3.0x |
| PVH5-IgA2 | 32.29 ± 0.53 | 11.36 ± 0.02 | 36.57 ± 0.18 | 7.21 ± 0.10 | 25.93 ± 0.34 | 18.70 ± 0.09 | ~4.4x | ~2.2x | ~1.9x |
| PVH6-IgA2 | 28.76 ± 0.11 | 4.62 ± 0.02 | 13.18 ± 0.02 | 6.82 ± 0.04 | 11.25 ± 0.06 | 7.67 ± 0.03 | ~4.2x | ~2.4x | ~1.7x |
| PVH7-IgA2 | 16.24 ± 0.08 | 8.42 ± 0.04 | 13.61 ± 0.03 | 2.95 ± 0.02 | 21.83 ± 0.12 | 6.44 ± 0.03 | ~5.5x | ~2.5x | ~2.1x |
| HVH1-IgA1 | 1.16 ± 0.02 | 10.64 ± 0.05 | 1.24 ± 0.02 | 0.10 ± 0.01 | 39.20 ± 0.25 | 0.42 ± 0.03 | ~11.6x | ~3.6x | ~2.9x |
| HVH2-IgA1 | PR | | | 0.83 ± 0.01 | 37.87 ± 0.25 | 3.13 ± 0.03 | NIL | | |
| HVH3-IgA1 | 5.60 ± 0.04 | 7.26 ± 0.04 | 4.06 ± 0.03 | 0.35 ± 0.01 | 39.68 ± 0.23 | 1.38 ± 0.03 | ~16.0x | ~5.4x | ~2.9x |
| HVH4-IgA1 | PR | | | 0.50 ± 0.01 | 38.45 ± 0.24 | 1.90 ± 0.03 | NIL | | |
| HVH5-IgA1 | 4.85 ± 0.04 | 7.52 ± 0.04 | 3.64 ± 0.03 | 0.62 ± 0.01 | 38.27 ± 0.22 | 2.37 ± 0.03 | ~7.8x | ~5.0x | ~1.5x |
| HVH6-IgA1 | 4.52 ± 0.04 | 7.40 ± 0.04 | 3.34 ± 0.02 | 0.43 ± 0.01 | 39.29 ± 0.22 | 1.71 ± 0.03 | ~10.5x | ~5.3x | ~1.9x |
| HVH7-IgA1 | 5.72 ± 0.04 | 6.86 ± 0.03 | 3.91 ± 0.02 | 0.57 ± 0.01 | 34.24 ± 0.19 | 1.96 ± 0.03 | ~10.0x | ~4.9x | ~1.9x |
| HVH1-IgA2 | PR | | | 0.61 ± 0.01 | 38.35 ± 0.24 | 2.33 ± 0.03 | NIL | | |
| HVH2-IgA2 | PR | | | 1.02 ± 0.02 | 34.01 ± 0.25 | 3.09 ± 0.04 | NIL | | |
| HVH3-IgA2 | 5.85 ± 0.05 | 7.42 ± 0.04 | 4.34 ± 0.03 | 0.34 ± 0.01 | 40.43 ± 0.24 | 1.38 ± 0.03 | ~17.2x | ~5.4x | ~3.1x |
| HVH4-IgA2 | PR | | | 0.99 ± 0.02 | 31.53 ± 0.25 | 3.10 ± 0.04 | NIL | | |
| HVH5-IgA2 | 5.23 ± 0.05 | 7.50 ± 0.05 | 3.86 ± 0.03 | 0.57 ± 0.01 | 36.56 ± 0.24 | 2.10 ± 0.03 | ~9.1x | ~4.8x | ~1.8x |
| HVH6-IgA2 | PR | | | 0.29 ± 0.01 | 39.23 ± 0.26 | 1.18 ± 0.03 | NIL | | |
| HVH7-IgA2 | 5.53 ± 0.05 | 7.26 ± 0.04 | 4.02 ± 0.03 | 0.59 ± 0.01 | 36.25 ± 0.23 | 2.17 ± 0.03 | ~9.3x | ~4.9x | ~1.8x |

**Supplementary Table S2**. BLI measurements of immobilized proteins G, L and A interacting with Pertuzumab (PVH) and Trastuzumab (HVH) 1-7 IgA1 and IgA2. The KD, ka and kd values with standard error of each variant are shown accordingly. Poor response (PR) indicates that the antibody construct did not yield reliable ka and kd measurements (in triplicates). All measurements were performed in triplicates and rounded off to the nearest 2 decimal places.

| Binding measurement to Superantigen | | | | | | | | | |
| --- | --- | --- | --- | --- | --- | --- | --- | --- | --- |
| Construct | Protein G Immobilization | | | Protein L Immobilization | | | Protein A Immobilization | | |
|  | KD (10^-9^) | ka (10^4^) | kd (10^-4^) | KD (10^-10^) | ka (10^4^) | kd (10^-4^) | KD (10^-8^) | ka (10^4^) | kd (10^-4^) |
| PVH1-IgA1 | 5.25 ± 0.05 | 1.78 ± 0.01 | 0.93 ± 0.01 | 17.94 ± 0.39 | 13.21 ± 0.06 | 2.37 ± 0.05 | PR | | |
| PVH2-IgA1 | 3.24 ± 0.03 | 3.09 ± 0.01 | 1.00 ± 0.01 | 20.82 ± 0.32 | 14.14 ± 0.05 | 2.94 ± 0.04 | 22.74 ± 0.46 | 0.76 ± 0.01 | 17.16 ± 0.06 |
| PVH3-IgA1 | 9.49 ± 0.13 | 0.92 ± 0.01 | 0.88 ± 0.01 | 7.95 ± 0.37 | 14.19 ± 0.06 | 1.13 ± 0.05 | 6.17 ± 0.13 | 2.62 ± 0.05 | 16.18 ± 0.17 |
| PVH4-IgA1 | 3.45 ± 0.03 | 2.81 ± 0.01 | 0.97 ± 0.01 | 14.45 ± 0.32 | 14.28 ± 0.06 | 2.06 ± 0.04 | 21.86 ± 0.45 | 0.81 ± 0.02 | 17.22 ± 0.06 |
| PVH5-IgA1 | 2.78 ± 0.03 | 3.71 ± 0.01 | 1.03 ± 0.01 | 28.92 ± 0.31 | 15.47 ± 0.06 | 4.46 ± 0.04 | 22.37 ± 0.47 | 0.82 ± 0.02 | 17.57 ± 0.06 |
| PVH6-IgA1 | 2.60 ± 0.03 | 4.12 ± 0.01 | 1.07 ± 0.01 | 24.70 ± 0.29 | 13.15 ± 0.04 | 3.25 ± 0.04 | 21.03 ± 0.41 | 0.93 ± 0.02 | 19.13 ± 0.07 |
| PVH7-IgA1 | 4.25 ± 0.04 | 1.92 ± 0.01 | 0.81 ± 0.01 | 22.64 ± 0.33 | 15.70 ± 0.07 | 3.56 ± 0.05 | PR | | |
| PVH1-IgA2 | 7.62 ± 0.06 | 1.07 ± 0.01 | 0.80 ± 0.01 | 11.56 ± 0.25 | 19.70 ± 0.09 | 2.27 ± 0.05 | PR | | |
| PVH2-IgA2 | 3.15 ± 0.02 | 3.00 ± 0.01 | 0.93 ± 0.01 | 31.00 ± 0.18 | 16.51 ± 0.04 | 5.06 ± 0.03 | 27.42 ± 0.78 | 0.61 ± 0.02 | 15.89 ± 0.06 |
| PVH3-IgA2 | 14.45 ± 0.16 | 0.71 ± 0.01 | 1.00 ± 0.01 | 5.21 ± 0.24 | 16.43 ± 0.06 | 0.85 ± 0.04 | 6.10 ± 0.1 | 3.20 ± 0.04 | 19.43 ± 0.14 |
| PVH4-IgA2 | 6.52 ± 0.05 | 1.23 ± 0.01 | 0.77 ± 0.01 | 11.4 ± 0.25 | 12.85 ± 0.03 | 1.46 ± 0.03 | PR | | |
| PVH5-IgA2 | 3.77 ± 0.02 | 2.66 ± 0.01 | 0.99 ± 0.01 | 22.66 ± 0.18 | 16.68 ± 0.04 | 3.78 ± 0.03 | 75.49 ± 6.00 | 0.23 ± 0.02 | 17.36 ± 0.07 |
| PVH6-IgA2 | 3.70 ± 0.02 | 2.54 ± 0.01 | 0.93 ± 0.01 | 21.69 ± 0.18 | 15.08 ± 0.03 | 3.26 ± 0.03 | 79.10 ± 10.68 | 0.44 ± 0.02 | 17.69 ± 0.06 |
| PVH7-IgA2 | 9.87 ± 0.10 | 0.85 ± 0.01 | 0.84 ± 0.01 | 12.93 ± 0.27 | 15.23 ± 0.05 | 1.95 ± 0.04 | PR | | |
| HVH1-IgA1 | 1.98 ± 0.04 | 6.79 ± 0.02 | 1.32 ± 0.03 | 48.38 ± 0.23 | 15.80 ± 0.04 | 7.64 ± 0.03 | 11.2 ± 0.11 | 1.65 ± 0.01 | 17.91 ± 0.06 |
| HVH2-IgA1 | 1.59 ± 0.04 | 9.07 ± 0.03 | 1.44 ± 0.04 | 88.53 ± 0.41 | 7.38 ± 0.02 | 6.52 ± 0.03 | 3.70 ± 0.02 | 1.90 ± 0.01 | 7.03 ± 0.03 |
| HVH3-IgA1 | 18.22 ± 0.28 | 0.83 ± 0.01 | 1.58 ± 0.02 | 1.35 ± 0.39 | 15.66 ± 0.09 | 0.21 ± 0.06 | 0.47 ± 0.01 | 37.13 ± 0.39 | 17.32 ± 0.12 |
| HVH4-IgA1 | 1.52 ± 0.04 | 10.79 ± 0.04 | 1.63 ± 0.04 | 51.31 ± 0.42 | 9.19 ± 0.03 | 4.53 ± 0.04 | PR | | |
| HVH5-IgA1 | 3.19 ± 0.04 | 3.93 ± 0.01 | 1.23 ± 0.02 | 10.82 ± 0.47 | 11.09 ± 0.05 | 1.20 ± 0.05 | 16.5 ± 0.33 | 0.82 ± 0.01 | 12.62 ± 0.05 |
| HVH6-IgA1 | 1.85 ± 0.04 | 8.48 ± 0.02 | 1.55 ± 0.03 | 18.6 ± 0.37 | 13.1 ± 0.05 | 2.43 ± 0.05 | 6.20 ± 0.10 | 3.33 ± 0.05 | 20.65 ± 0.15 |
| HVH7-IgA1 | 2.52 ± 0.04 | 5.32 ± 0.01 | 1.32 ± 0.02 | 11.55 ± 0.42 | 13.82 ± 0.07 | 1.59 ± 0.06 | 3.43 ± 0.06 | 3.52 ± 0.05 | 12.00 ± 0.13 |
| HVH1-IgA2 | 2.51 ± 0.06 | 6.56 ± 0.02 | 1.48 ± 0.04 | 47.05 ± 0.59 | 5.04 ± 0.01 | 2.26 ± 0.02 | 0.28 ± 0.01 | 3.90 ± 0.01 | 0.96 ± 0.02 |
| HVH2-IgA2 | 1.70 ± 0.03 | 10.28 ± 0.03 | 1.58 ± 0.04 | 56.36 ± 0.45 | 7.29 ± 0.02 | 4.00 ± 0.03 | 0.84 ± 0.01 | 3.33 ± 0.01 | 2.91 ± 0.03 |
| HVH3-IgA2 | 14.12 ± 0.24 | 0.68 ± 0.01 | 0.96 ± 0.01 | 0.07 ± 0.25 | 19.63 ± 0.09 | 0.01 ± 0.05 | 0.42 ± 0.01 | 37.44 ± 0.34 | 15.18 ± 0.10 |
| HVH4-IgA2 | 1.79 ± 0.04 | 7.92 ± 0.02 | 1.38 ± 0.03 | 72.59 ± 0.42 | 5.78 ± 0.01 | 4.25 ± 0.02 | 7.77 ± 0.09 | 1.39 ± 0.01 | 11.15 ± 0.05 |
| HVH5-IgA2 | 4.14 ± 0.04 | 3.32 ± 0.01 | 1.29 ± 0.01 | 1.91 ± 0.20 | 20.10 ± 0.07 | 0.36 ± 0.04 | 27.42 ± 0.91 | 0.58 ± 0.02 | 14.70 ± 0.06 |
| HVH6-IgA2 | 1.26 ± 0.03 | 13.16 ± 0.05 | 1.64 ± 0.04 | 42.05 ± 0.25 | 14.09 ± 0.04 | 5.86 ± 0.03 | 5.02 ± 0.04 | 3.20 ± 0.02 | 16.6 ± 0.07 |
| HVH7-IgA2 | 3.06 ± 0.03 | 4.16 ± 0.01 | 1.26 ± 0.01 | 7.06 ± 0.25 | 15.83 ± 0.05 | 1.08 ± 0.04 | 4.30 ± 0.08 | 6.29 ± 0.10 | 19.39 ± 0.19 |

**Supplementary Table S3**: Reported non-binding and binding of protein G and A to IgA from various product specification sheets. <https://www.abcam.com/kits/antibody-binding-affinities-of-protein-a-protein-g-protein-l-and-jacalin>; <https://www.sigmaaldrich.com/SG/en/technical-documents/technical-article/protein-biology/protein-pulldown/protein-a-g-binding>; <https://www.neb.sg/tools-and-resources/selection-charts/affinity-of-protein-ag-for-igg-types-from-different-species>; <https://www.bio-rad-antibodies.com/binding-affinities.html>; <https://cdn.cytivalifesciences.com/dmm3bwsv3/AssetStream.aspx?mediaformatid=10061&destinationid=10016&assetid=19097>; <https://www.agilent.com/cs/library/applications/5991-6094EN.pdf>)

| **Superantigens Binding Strength to IgA** | | |
| --- | --- | --- |
| **Companies** | **Protein G** | **Protein A** |
| Abcam | No Binding | Weak Binding |
| Sigma Aldrich | No Binding | Variable Binding |
| NEB | No Binding | No Binding |
| Biorad | No Binding | Weak Binding |
| Cytiva | No Binding | Variable Binding |
| Aligent | No Binding | Weak Binding |

**References**

1 Lyskov, S. *et al.* Serverification of Molecular Modeling Applications: The Rosetta Online Server That Includes Everyone (ROSIE). *PLOS ONE* **8**, e63906, doi:10.1371/journal.pone.0063906 (2013).

2 Weitzner, B. D. *et al.* Modeling and docking of antibody structures with Rosetta. *Nature Protocols* **12**, 401-416, doi:10.1038/nprot.2016.180 (2017).

3 Su, C. T.-T., Lua, W.-H., Ling, W.-L. & Gan, S. K.-E. Allosteric Effects between the Antibody Constant and Variable Regions: A Study of IgA Fc Mutations on Antigen Binding. *Antibodies* **7**, 20 (2018).

4 Royle, L. *et al.* Secretory IgA <em>N</em>- and <em>O</em>-Glycans Provide a Link between the Innate and Adaptive Immune Systems *. *Journal of Biological Chemistry* **278**, 20140-20153, doi:10.1074/jbc.M301436200 (2003).

5 Ohyama, Y. *et al.* Analysis of O-glycoforms of the IgA1 hinge region by sequential deglycosylation. *Scientific Reports* **10**, 671, doi:10.1038/s41598-020-57510-z (2020).

6 Park, S.-J. *et al.* CHARMM-GUI Glycan Modeler for modeling and simulation of carbohydrates and glycoconjugates. *Glycobiology* **29**, 320-331, doi:10.1093/glycob/cwz003 (2019).

7 Mark Abraham, Berk Hess, David van der Spoel & Lindahl, E. GROMACS 2019.3 Manual. *Zenodo*, doi:10.5281/ZENODO.3243834 (2019).

8 van Zundert, G. C. P. *et al.* The HADDOCK2.2 Web Server: User-Friendly Integrative Modeling of Biomolecular Complexes. *Journal of Molecular Biology* **428**, 720-725, doi:<https://doi.org/10.1016/j.jmb.2015.09.014> (2016).

9 Posgai, M. T. *et al.* FcαRI binding at the IgA1 C<sub>H</sub>2–C<sub>H</sub>3 interface induces long-range conformational changes that are transmitted to the hinge region. *Proceedings of the National Academy of Sciences* **115**, E8882-E8891, doi:10.1073/pnas.1807478115 (2018).

10 Herr, A. B., Ballister, E. R. & Bjorkman, P. J. Insights into IgA-mediated immune responses from the crystal structures of human FcαRI and its complex with IgA1-Fc. *Nature* **423**, 614-620, doi:10.1038/nature01685 (2003).

11 Nadalin, F. & Carbone, A. Protein–protein interaction specificity is captured by contact preferences and interface composition. *Bioinformatics* **34**, 459-468, doi:10.1093/bioinformatics/btx584 (2017).

12 Schrodinger, LLC. *The PyMOL Molecular Graphics System, Version 1.8* (2015).

13 Grant, B. J., Rodrigues, A. P., ElSawy, K. M., McCammon, J. A. & Caves, L. S. Bio3d: an R package for the comparative analysis of protein structures. *Bioinformatics* **22**, 2695-2696, doi:10.1093/bioinformatics/btl461 (2006).
